# Disruption of neutrophil homeostasis is associated with functional alterations in mitochondria of critically ill COVID−19 patients

**DOI:** 10.1038/s41598-026-38741-y

**Published:** 2026-03-01

**Authors:** Aya A. Elkhodiry, Basma A. Yasseen, Hajar El-sayed, Mona Zidan, Azza G. Kamel, Rehab Hamdy, Sara Gohar, Mohamed A. Badawy, Aya Saber, Hend E. El-Shqnqery, Omar Samir, Ahmed A. Sayed, Ashraf Eltaher, Hadeer Abdelkhalek, Mennatullah Eltaras, Malak W. ElBenhawi, Jantan Dawa, Marwa S. Hamza, Riem M. El-Messiery, Mohamed El Ansary, Engy A. Abdel-Rahman, Sameh S. Ali

**Affiliations:** 1 Research Department, Children’s Cancer Hospital Egypt 57357, Cairo, 11441 Egypt; 2 Department of Clinical Pharmacy Practice, Faculty of Pharmacy, The British University in Egypt, Cairo, Egypt; 3 Infectious Disease Unit, Internal Medicine Department, Faculty of Medicine, Cairo University, Cairo, Egypt; 4 Department of Intensive Care, Faculty of Medicine, Cairo University, Cairo, Egypt; 5 Pharmacology Department, Faculty of Medicine, Assuit University, Assuit, Egypt

**Keywords:** Neutrophilia, Critically-ill COVID−19 patients, Calcium, Apoptosis, Mitochondria, Biomarkers, Cell biology, Diseases, Immunology, Medical research, Molecular biology

## Abstract

Understanding the molecular mechanisms underlying neutrophil dynamics during COVID−19 disease progression is essential for managing severe inflammatory conditions. We investigated whether alterations in neutrophil mitochondrial function and calcium handling are associated with disrupted neutrophil homeostasis in critically ill COVID−19 patients. We analyzed neutrophil counts, phenotypes, and apoptotic profiles in critically ill COVID−19 survivors (ICU-S) and non-survivors (ICU-NS) compared with healthy controls. Flow cytometry, metabolic profiling, immunofluorescence imaging, and small RNA sequencing (miRNA-seq) were used to characterize neutrophil apoptosis-related pathways and mitochondrial function in freshly isolated neutrophils. Critically ill COVID−19 patients showed marked neutrophilia and a higher proportion of immature CD16low neutrophils relative to controls. Both ICU-S and ICU-NS groups exhibited reduced neutrophil apoptosis, as evidenced by fewer annexin V+ cells and lower cleaved caspase−3 signal compared with healthy controls. Although exploratory miRNA-seq in a small subset of ICU patients identified differentially expressed miRNAs with predicted enrichment in apoptosis- and calcium-related pathways, these mortality-associated miRNA signatures were not corroborated by functional apoptosis readouts (cleaved caspase−3 and annexin V) between ICU-S and ICU-NS. Neutrophils from ICU patients also demonstrated altered calcium handling, hyperpolarized mitochondrial membrane potential, increased complex II–linked respiration, and elevated mitochondrial ROS relative to controls. Neutrophils from critically ill COVID−19 patients display coordinated alterations in calcium handling, mitochondrial activity, and apoptosis consistent with impaired neutrophil clearance and disrupted homeostasis. These findings are observational and do not establish causality; the miRNA results should be interpreted as hypothesis-generating rather than validated mortality biomarkers.

**Supplementary Information:**

The online version contains supplementary material available at 10.1038/s41598-026-38741-y.

## Introduction

The coronavirus disease 2019 (COVID-19) pandemic, caused by severe acute respiratory syndrome coronavirus 2 (SARS-CoV-2), has produced an enduring global burden of critical illness^1–4^. Severe COVID-19 is characterized by dysregulated inflammation and immune dysfunction, and a consistent clinical correlate of poor outcome is peripheral neutrophilia^2,5–8^. Neutrophils can limit infection through phagocytosis, degranulation, and reactive oxygen species production^9,10^, but excessive or prolonged activation can drive tissue injury and thrombo-inflammation^11,12^. In severe COVID-19, neutrophilia has been linked to organ injury^13^, hypercoagulability^14^, and inflammatory tissue migration with subsequent damage^15,16^, yet the mechanisms that promote sustained neutrophil accumulation in the most critically ill patients remain incompletely defined. Persistent neutrophil-associated immune responses have also been implicated in post-COVID-19 long-term pulmonary sequelae^17^.

Since the early phase of the pandemic, high-dimensional profiling studies have further characterized neutrophil heterogeneity, metabolic reprogramming, and mitochondrial dysfunction in COVID-19. Proteomic and metabolomic analyses reveal widespread alterations in neutrophil glycolysis, redox balance, and central carbon metabolism in severe COVID-19, alongside persistent activation signatures in convalescent patients^18,19^. Contemporary reviews also emphasize expansion of immature and low-density neutrophil subsets, exaggerated neutrophil extracellular trap (NET) formation, and sustained contributions to long COVID and post-acute sequelae^20–23^. These observations indicate that neutrophil state transitions and persistence are likely central to pathogenesis, but the cellular pathways that regulate neutrophil survival and clearance in critical COVID-19 remain an important gap.

Neutrophil homeostasis, the balance between neutrophil production and elimination, is regulated by granulopoiesis in the bone marrow and clearance mechanisms involving apoptosis followed by phagocytosis^24,25^. This balance supports immune defense while preventing pathological accumulation. Calcium and mitochondria are pivotal regulators of key neutrophil functions including chemotaxis, phagocytosis, activation, cytokine release, and apoptosis^26–29^. Calcium influx and homeostasis play dual roles in the immune response, promoting both initiation and resolution^30,31^. Previous studies have concluded that increased cytosolic calcium can be pro-apoptotic by sensitizing the mitochondrial transition towards apoptosis, ultimately leading to cell death^32,33^. Additionally, mitochondrial calcium overload can trigger apoptosis by causing mitochondrial swelling, rupture of the outer membrane, and release of apoptotic factors^34^.

Physiologically, mitochondria have traditionally been regarded as regulators of neutrophil apoptosis with minimal contribution to energy production^35^. However, recent evidence highlights broader mitochondrial roles in neutrophils despite glycolysis remaining the dominant energy pathway [reviewed in^27^. For example, mitochondria contribute to NET formation^36,37^, and regulate adhesion, intravascular crawling, and transendothelial migration, linking mitochondrial pathways to neutrophil development and survival^38^. In parallel, the concept of ‘viral mitochondriopathy’ proposes that SARS-CoV-2–associated mitochondrial dysfunction can contribute to prolonged immunometabolic dysregulation across tissues^39–42^. Situating neutrophils within this framework may help explain how systemic mitochondrial perturbations interact with neutrophil homeostasis in severe COVID-19.

In the context of COVID-19, the roles of calcium and mitochondria in neutrophil dynamics and function remain poorly understood, with existing reports being both scarce and conflicting. Studies examining calcium levels in COVID-19 patients have found that hypocalcemia in hospitalized individuals is associated with worse clinical outcomes and increased disease severity^43–46^. Additionally, some studies have reported increased glycolytic activity in neutrophils from COVID-19 patients^47,48^, whereas others report upregulation of oxidative phosphorylation genes^49^. These paradoxical findings, together with the predictive value of neutrophil proportions and neutrophil-to-lymphocyte ratio (NLR) for mortality^6,8,22,43,50–56^, motivated a deeper exploration of mitochondrial and calcium-linked pathways in neutrophils from the most severe cases. In our related series of papers, we demonstrated downstream pathological implications of neutrophilia including oxidative stress and protein damage^57^, neutrophil and platelet activation and hypercoagulability^14^, as well as hyperlactatemia and metabolic blood acidosis affecting oxygen delivery^58^. In the current work we attempt to shed light on relevant upstream molecular and cellular factors associated with neutrophilia in critically ill patients.

## Materials and methods

### Study design and participants

This research aimed to assess the impact of COVID−19 severity on neutrophil homeostasis and functional activity, comparing individuals with severe COVID−19 to healthy controls. The study was structured as a prospective observational cohort analysis, enrolling patients with confirmed RT-PCR-positive COVID−19. Cases of severe COVID−19 were identified using nasopharyngeal swab RT-PCR results along with lung CT scans. Participants were recruited from the ICU facility at Internal Medicine Quarantine Hospital, Kasr Alainy Cairo University Hospital.

Standard supportive treatment, including supplemental oxygen and symptomatic care, was provided to all patients. Those experiencing moderate-to-severe hypoxia, characterized by a need for a fraction of inspired oxygen (FiO2) of 40% or higher, were transferred to the intensive care unit (ICU) for further management that might include invasive mechanical ventilation when deemed necessary. The study cohort comprised patients admitted to ICU, categorized into two cohorts based on survival outcomes: ICU survivors (ICU-S) and ICU non-survivors (ICU-NS). Inclusion criteria were: (1) adults aged 18 years or older; (2) RT-PCR-confirmed SARS-CoV−2 infection; (3) severe COVID−19 requiring ICU admission with fraction of inspired oxygen (FiO2) of 40% or higher. Exclusion criteria were: (1) mild or moderate COVID−19 symptoms not requiring ICU admission; (2) respiratory distress with negative SARS-CoV−2 PCR result; (3) inability to provide informed consent. Controls were recruited from hospital staff and the local community during the same period as patient recruitment.

Due to the lack of prior reported studies on neutrophil homeostasis in COVID−19 patients at the time of recruitment, a formal statistical method to determine the required sample size was not employed. Instead, the sample size was based on sample availability, leading to a total inclusion of 94 COVID−19 patients (*N* = 94) from October 4, 2020, to December 7, 2021. Over the course of follow-up period, 58 patients died. All collected samples were investigated by blinded operators and were included in the final analysis. Written informed consent was obtained from all the participants. The study adhered to the Declaration of Helsinki (2013 revision, Fortaleza) and received approval from the Institutional Review Board of the Children’s Cancer Hospital Egypt 57,357 (protocol code: 31−2020, initial approval on July 6, 2020, renewed on July 28, 2021). The Children’s Cancer Hospital 57,357 Research Department served as the coordinating laboratory where all sample processing and analyses were conducted; patient recruitment and blood collection occurred at Kasr Alainy Cairo University Hospital’s ICU following that institution’s clinical protocols for COVID−19 research.

### Blood samples collection, handling, and processing

Fresh blood samples were collected from all participants in Acid Citrate Dextrose (ACD) tubes (Greiner Bio-One GmbH, Kremsmünster, Austria). Samples were collected within 24–48 h of ICU admission, following initial clinical stabilization. For patients with extended ICU stays, samples were obtained during the first 72 h of admission to capture the acute phase of critical illness. For flow cytometry measurements, whole blood was incubated with RBCs lysis buffer composed of NH_4_Cl (ammonium chloride), NaHCO_3_ (sodium bicarbonate), and EDTA (disodium) for 15 min^59^. For neutrophils isolation, the remaining 8 mL whole blood was processed as previously described^60^. Briefly, whole blood was centrifuged at 300×g at 25 °C for 15 min without applying brakes. The platelet-rich plasma layer, located at the top, was transferred to a new polypropylene tube. The remaining lower layer, containing white blood cells, red blood cells, and plasma, was gently layered over an equal volume of the lymphocyte separation medium (1.077). The tubes were centrifuged at 500×g for 35 min at 25 °C without applying brakes and with reduced acceleration. Following centrifugation, the upper three layers were removed, and the neutrophil-enriched layer was collected and diluted in 10 ml Hank’s Balanced Salt Solution (HBSS), and then centrifuged again at 350×g for 10 min at 25 °C to form a pellet. The pellet was resuspended in RBCs lysis buffer and incubated at room temperature for 15 min, and centrifuged for 10 min at 350×g. The final pellet was resuspended in 100 µL phosphate-buffered saline (PBS). Neutrophils were manually counted using a hemocytometer. Cell viability was assessed for each sample via trypan blue exclusion, and only samples demonstrating more than 90% viability were included in subsequent analyses^57,60^.

### Assessment of neutrophil counts and neutrophils maturity by flow cytometry

As mentioned previously, for flow cytometry measurements, whole blood was incubated with RBCs lysis buffer composed of NH_4_Cl (ammonium chloride), NaHCO_3_ (sodium bicarbonate), and EDTA (disodium) for 15 min^59^. The resuspended lysed whole blood cells were centrifuged at 500xg for 5 min and washed with 1x PBS. The cell pellet was resuspended in PBS, and neutrophil detection was carried out using a CytoFLEX V5-B5-R3 flow cytometer equipped with 13 detectors and three lasers (Beckman Coulter Life Sciences CytoFLEX benchtop flow cytometer). The cell suspension containing all blood cells except RBCs was incubated in the dark at room temperature for 30 min at the manufacturer’s recommended concentrations with CD66b-APC-Alexa Fluor 750 (Beckman Coulter Life Sciences, B08756) and CD16-FITC (Beckman Coulter Life Sciences, 6604894) for the detection of neutrophils and neutrophil subsets. The cells were then washed with 1x PBS and suspended in 300 µl PBS. In total, 10,000 events were acquired and recorded for each sample. The data were analyzed using CytExpert v2.3 software to assess the percentage and mean fluorescence intensities (MFIs) of cellular subsets. Neutrophils were identified using the neutrophil-specific positive marker “CD66b” for gating. Neutrophil maturity was evaluated based on the expression of CD16 within the CD66b-positive population, where (CD66b^+^CD16^hi^) indicates a mature neutrophil population and (CD66b^+^CD16^lo^) indicates an immature neutrophil population. For each stained sample, the corresponding negative control sample was used throughout the experiments.

### Assessment of neutrophils apoptosis by flow cytometry

Neutrophils were stained with CD66b-APC-Alexa Fluor 750 (Beckman Coulter Life Sciences, B08756, USA) as described previously. The cells were washed with 1x PBS and resuspended in 50 µL of 1X annexin binding buffer containing 2 µL of FITC-annexin V (Miltenyi Biotec, 130−092−052) or 2 µL Annexin V Pacific Blue (Thermo Fischer, A35122) according to the availability to detect early apoptosis (Annexin V^+^ cells). The cells were incubated in the dark at room temperature for 15 min. After incubation, the cells were washed with 1 mL 1X annexin binding buffer and suspended in 300 µL of 1X annexin binding buffer for acquisition. In total, 10,000 events were acquired and recorded for each sample. Data were analyzed using CytExpert v2.3 software to assess the percentage of early apoptotic cells (Annexin V+ cells). For each stained sample, the corresponding negative control sample was used throughout the experiments.

### Measurement of apoptosis in isolated neutrophils by fluorescence imaging

Apoptosis in isolated neutrophils was detected and quantified using annexin V dye (Thermo Fisher Scientific, A23202). Neutrophils were plated at a density of 100,000 cells/well and allowed to settle and adhere to polystyrene 96-well plate for 30 min at 37 °C. The plates were then centrifuged to form a monolayer. The cells were then stained with Annexin V in 1x annexin binding buffer for 30 min, according to the manufacturer’s recommendations. Images were acquired (1–5 images per subject) using the Cytation 5 Cell Imaging Multi-mode Reader (Agilent Technologies). Identical acquisition parameters (exposure, gain, filters, objective) were used across groups on a Cytation 5. Gen5 (v3.08) applied standardized background subtraction. Per-cell mean fluorescence intensity was normalized to DAPI to control for minor variation in cell adherence/focus.

### Small RNA library preparation and sequencing

RNA libraries were generated using the NEXTFLEX Small RNA-Seq Kit (PerkinElmer, USA; Cat. No.: NOVA−5132−05). For each library, 400 ng of purified RNA was used as an input for library preparation according to the manufacturer’s instructions. The ligated libraries were reverse-transcribed and amplified using unique barcode primers for each library. DNA fragments of approximately 150 bp (miRNA sequences plus 3′ and 5′ adaptors) were determined using 6% TBE-PAGE and then retrieved in a 300 µl of elution buffer for purification. The size distribution of the pooled libraries was analyzed using a Bioanalyzer DNA assay (Agilent, USA; Cat. No.: 5067−1504) and the concentration was assessed using the Qubit dsDNA HS Assay (ThermoFisher Scientific, USA; Cat. No.: Q33230). The final pooled library was sequenced for approximately 2,693 miRNAs using the Illumina MiSeq system (Illumina, Inc., USA).

### Bioinformatics analysis

Raw data quality was assessed using FastQC, and low-quality reads and adaptors were trimmed using Cutadapt following the manufacturer’s guidelines. The filtered reads were mapped to the human genome reference GRCh38 using Bowtie 1 (accession number: GCA_000001405.29). Mapped reads were quantified using FeatureCounts based on the human miRNA coordinate file (gff) retrieved from miRBase database release 22.1. Count data were filtered and normalized, and differential expression analysis between survivor and non-survivor groups was performed using the DESeq2 package in R V4.1.2. Average sequencing depth was 30.98×, and the mean mapping rate to miRBase was 54.42% across all samples. Differentially expressed miRNAs (DEMs) were selected to have adjusted p-value (Adj. p) value < 0.05. The log2 fold changes and adjusted p-values of the DEMs were visualized using a lollipop plot created using the ggplot R package.

### Identification of miRNA putative gene targets and network analysis

Pathway enrichment analysis for differentially expressed miRNAs was performed using the MirCarta database against the KEGG database, and only pathways supported by experimental evidence were selected. Enriched pathways were retrieved, and their visualization was conducted using the Pathview R package to represent regulated genes^61–63^.

### Measurement of cyclophilin D, MICU1, and cleaved caspase 3 in isolated neutrophils by fluorescent imaging

The expression levels of the calcium regulators cyclophilin D and MICU1 in the isolated neutrophils were assessed. The seeded cells were incubated for 30 min at 37 °C in order to settle and adhere to polystyrene 96-well plate followed by centrifugation to form a monolayer. The cells were fixed with 4% PFA for 15 min, washed with PBS, and permeabilized with 0.1% Triton-X for 15 min, followed by another PBS wash step. The cells were then blocked with 5% Bovine Serum Albumin (BSA) for 1 h and stained with the specific primary antibodies rabbit cyclophilin D (Cyclophilin 40 antibody, GenTex, GTX104038; 1:100), Rabbit MICU1 (Anti-CBARA1 antibody (MICU1), Abcam, ab102830; 1:100), and rabbit cleaved caspase 3 (CC3, Cell Signaling, 9661 S; 1:100) overnight at 4 °C. The plates were washed twice with PBS and then stained with the appropriate secondary antibody, Goat Anti-Rabbit IgG (H + L) Cross-Adsorbed Secondary Antibody, Alexa Fluor 594 (Invitrogen, A−11012) for cyclophilin D and cleaved caspase 3 and Anti-Rabbit IgG (H + L), F(ab’)2 fragment (Alexa Fluor 488 Conjugate), (Cell Signaling, 4412 S) for MICU1 at a concentration of 1:400 for all conditions for 1 h at room temperature. The cells were then washed and stained with DAPI (Hoechst 33342 solution, Thermo Fisher Scientific, 62249) at a final concentration of 10 µM in PBS for 30 min at 37 °C. A Cytation 5 Cell Imaging Multi-Mode Reader (BioTek) was used to acquire images on a 20X lens using the proper fluorescence filter cubes λ_ex_=469 ± 17 nm and λ_em_=525 ± 19 nm for the GFP channel and λ_ex_=586 ± 7 nm and λ_em_=647 ± 28 nm for the Texas Red channel acquiring to 2–4 images per subject. All fluorescence images were acquired on Cytation 5 using identical exposure, gain, and filter settings; Gen5 performed standardized background sutraction prior to quantification. The Gen5 3.08 Imager (software) processed the images to quantify the fluorescence intensity. Per-cell mean fluorescence intensity was normalized to DAPI to control for minor variation in cell adherence/focus.

### Measurement of intracellular calcium in isolated neutrophils by fluorescent imaging

Intracellular calcium levels in isolated neutrophils were assessed using Fluo−4-AM, a cell permeant (Fluo−4-AM, Thermo Fisher Scientific, F14201). The seeded cells were incubated for 30 min at 37 °C in order to settle and adhere to polystyrene 96-well plate followed by centrifugation to form a monolayer. The cells were stained with Fluo−4-AM (10 µM) in the presence of Pluronic F−127 (0.02%) in HEPES buffer for 30 min. After staining, the cells were washed and stained with DAPI (Hoechst 33342 solution, Thermo Fischer Scientific, 62249) at a final concentration of 10 µM in PBS for 30 min at 37 °C. Images were acquired using a Cytation 5 Cell Imaging Multi-Mode Reader (BioTek) on a 20X lens using proper fluorescence filter cubes (λ_ex_=469 ± 17 nm and λ_em_=525 ± 19 nm), acquiring 2–4 images per subject. All fluorescence images were acquired on Cytation 5 using identical exposure, gain, and filter settings; Gen5 performed standardized background subtraction prior to quantification. The Gen5 3.08 Imager (software) processed the images to quantify the fluorescence intensity. Per cell mean fluorescence intensity was normalized to DAPI to control for minor variation in cell adherence/focus.

### Measurement of intracellular calcium by flow cytometry

Intracellular calcium levels in different cell populations were measured in whole blood samples using Fluo−4-AM, a cell permeant (Fluo−4-AM, Thermo Fisher Scientific, F14201). Suspended cells were incubated with Fluo−4-AM (10 µM) in the presence of Pluronic F−127 (0.02%) for 15 min, followed by the addition of a combination of monoclonal antibodies, including CD66b-APC-Alexa Fluor 750 (for neutrophils), in HEPES buffer for 15 min at room temperature in the dark. The cells were then washed, suspended in 300 µl of HEPES buffer, and incubated for 20 min. A total of 10,000 events were recorded and analyzed using the CytExpert program.

### Measurement of transmembrane potential by flow cytometry

The transmembrane potentials of different cell populations were measured in whole blood samples using tetramethylrhodamine methyl ester perchlorate (TMRM; Sigma, T5428). Suspended cells were incubated with TMRM (1 µM) and an antibody against CD66b-APC-Alexa Fluor 750 (for neutrophils) for 30 min at room temperature in the dark. The cells were then washed with PBS and suspended in 300 µl of PBS. A total of 10,000 events were recorded and analyzed using the CytExpert program.

### Measurement of oxygen consumption rate (OCR) in neutrophils by seahorse XF96 flux analyzer

The oxygen Consumption Rate (OCR) of neutrophils was assessed using an Agilent Seahorse XF analyzer as described previously^14^. Neutrophils were plated in 96-well plates at a density of 2 × 10^5^ cells/well in unbuffered Dulbecco’s Modified Eagle’s medium (DMEM; with 1 mM pyruvate, 5.5 mM D-glucose, and 4 mM L-glutamine, pH 7.4, at 37 °C). The plates were centrifuged at 800xg for 5 minutes to form monolayer in the wells and then incubated for 30–40 min at 37 °C in a non-CO2 incubator. A baseline measurement of OCR was recorded for 30 min. The mitochondrial stress test was subsequently performed by measuring OCR values following injections of oligomycin (2.5 µM), carbonyl cyanide p-(trifluoro-methoxy) phenyl-hydrazone (FCCP) (2 µM), and Rotenone/Antimycin A mixture (Rot/AA) (2.5 µM). All OCR values were normalized to the initial seeding cell density. Maximal mitochondrial respiration was calculated by subtracting non-mitochondrial oxygen consumption from the average rate after the addition of FCCP.

### Measurements of mitochondrial respiratory rates by oxygraph−2K

Mitochondrial respiratory function was assessed at 37°C using a high-resolution respirometry system, Oxygraph−2K (Oroboros Instruments, Innsbruck, Austria), as previously described^60^. Prior to starting the experiment, oxygen calibration was performed by allowing the respiration medium (MIR05) to equilibrate with air in the oxygraph chambers until a stable signal was detected. Neutrophils were then transferred into the chambers and permeabilization was achieved by adding 50 µg/ml saponin. The 50 µg/ml saponin concentration for neutrophil permeabilization in high-resolution respirometry follows established protocols for immune cells and was optimized to achieve selective plasma membrane permeabilization whilst preserving mitochondrial outer membrane integrity. This has been verified by adding external cytochrome C while monitoring changes in oxygen consumption rate. Saponin concentrations employed didn’t elicit significant increase in oxygen consumption rate thus confirming intact mitochondrial outer membrane as shown in Supplementary Fig. 5. The substrate–uncoupler–inhibitor titration (SUIT) protocol was applied as follows: pyruvate (5 mM), malate (5 mM), (PM) adenosine diphosphate (D, 1 mM), glutamate (G, 5 mM), succinate (S, 10 mM), oligomycin (O, 1 µg/mL) carbonyl cyanide−4-(trifluoromethoxy) phenylhydrazone (FCCP) (F, multiple 0.5 µM infusions), rotenone (R, 2 µM), Antimycin A (AA, 1.25 µM), N, N, N’, N’-tetramethyl-p-phenylenediamine (0.5 mM)/ Ascorbate (2 mM), (TMPD/Asc). The oxygen consumption rates were normalized to the neutrophil count and calculated as the negative time-derivative of the oxygen concentration. Mitochondrial respiratory parameters were assessed as follows: (1) Oxidative phosphorylation (OXPHOS) rate after adding saturating ADP in the presence of pyruvate, malate, and/or glutamate (OXPHOS-I) or in the presence of succinate as a complex II substrate (OXPHOS I + II). (2) Electron transfer system (ETS) capacity (maximum respiration rate) in the presence of FCCP. (3) Activity of Complex IV after the addition of TMPD/ASC. DatLab VR software version 7.4.0.4 (Oroboros Instruments, Innsbruck, Austria) was used for the data acquisition and analysis.

### Electron microscopy

Fresh peripheral citrated blood samples (10 mL) were centrifuged at 800xg for 5 min with no brakes. The top plasma layer was removed, leaving less than 1 ml above the buffy coat. Cold fixative containing 82 mM sodium monophosphate, 20 mM sodium hydroxide, 4% formaldehyde, and 0.01% glutaraldehyde (pH 7.2) was added dropwise to the sample and left for 30 min. The coat was then transferred to the same fixative for short-term storage at 4 °C. Fixed samples were processed for transmission electron microscopy (TEM), as described previously^64^. Ultrathin sections were post-stained in saturated uranyl acetate and lead citrate and then examined using a JSM1400 plus-JEOL TEM (Alexandria University microscopy unit)^14,65^. Images were analyzed using ImageJ software, where mitochondria were counted visually for each neutrophil, and the freehand tool was used to extract mitochondrial parameters, such as area and density (1–5 neutrophils per patient, 3–4 patients per group).

### Measurement of Mitotracker and MitoSOX in isolated neutrophils by fluorescent imaging

Mitochondrial density and mitochondrial reactive oxygen species (mROS) in isolated neutrophils were assessed using Mitotracker (Mitotracker Red FM, ThermoFischer Scientific, M22425) and MitoSOX (MitoSOX Mitochondrial Superoxide Indicators, ThermoFischer Scientific, M36008), respectively. Isolated cells were seeded and incubated for 30 min at 37 °C, to settle and adhere to polystyrene 96-well plate followed by centrifugation to form a monolayer. Cells were stained with MitoTracker at a final concentration of 0.5 µM in PBS or MitoSOX at a final concentration of 5 µM in HBSS for 30 min. The cells were then washed and stained with DAPI (Hoechst 33342 Solution, Thermo Fischer Scientific, 62249) at a final concentration of 10 µM in PBS for 30 min at 37 °C. A Cytation 5 Cell Imaging Multi-Mode Reader (BioTek) was used to acquire images on a 20X lens using the proper fluorescence filter cubes (λ_ex_=586 ± 7 nm and λ_em_=647 ± 28 nm for MitoTracker and λ_ex_= 531 ± 40 nm and λ_em_=593 ± 40 nm for MitoSOX) acquiring to 2–4 images per subject. All fluorescence images were acquired on Cytation 5 using identical exposure, gain, and filter settings; Gen5 performed standardized background subtraction prior to quantification. The Gen5 3.08 Imager (software) processed the images to quantify the fluorescence intensity. Per cell mean fluorescence intensity was normalized to DAPI to control for minor variation in cell adherence/focus.

### Statistical analysis

Statistical analyses and graphical representations were conducted using OriginPro 2021 software (OriginLab Corporation, Northampton, USA). Half-violin plots were used to outline statistical analysis outcomes and to demonstrate data scatter, distribution, and mean ± SD or SEM, as stated in the figure legends. The exact p-values are provided for each comparison in the majority of the graphs. Continuous variables are presented as means and standard deviations or medians (IQR). Normality of continuous variables was assessed using the Shapiro–Wilk test prior to statistical analysis, and either the t-test or Mann–Whitney test was used to compare continuous variables according to normality. For normally distributed variables, one-way ANOVA followed by Tukey’s post-hoc test was used to compare differences among the three groups, whereas non-normally distributed variables were analyzed using the Kruskal–Wallis test with Dunn’s post-hoc test. For categorical variables, the Chi-square test was applied, and Fisher’s exact test was used when the expected count was less than 5. A p-value of less than 0.05, indicated significant evidence of an association between mortality and the variable.

## Results

### Demographic, clinical, and laboratory hematologic characteristics of the studied COVID−19 patients

Supplementary Table 1 summarizes the demographic and clinical characteristics of the study patients, categorized into ICU-S, *n* = 36 and ICU-NS, *n* = 58. Diabetes was significantly less prevalent among ICU-S (16.7%) compared to ICU-NS (41.4%), with a p-value of 0.01. ICU-S patients had a slightly higher median oxygen saturation (sO_2_) of 94% versus 90% in ICU-NS, approaching statistical significance (*p* = 0.05). The use of remdesivir was notably more frequent among ICU-NS (25.8%) than ICU-S (8.3%) (*p* = 0.03). Other factors, including sex, age, cardiovascular disease, cancer, asthma, insulin use, anticoagulant use, steroids, hydroxychloroquine, IL−6 inhibitors, ivermectin, carbapenem antibiotics, fluoroquinolone, and oxazolidinone, showed no significant differences between groups. Healthy controls (*N* = 29) were adult volunteers (mean age 33.8 ± 9.3 years; 19 males/65.5%, 10 females/ 35.5%; age range 19–55 years) without acute illness, known chronic diseases (diabetes, cardiovascular disease, cancer, autoimmune conditions), immunosuppressive medication use, or recent infections. Clinical characteristics beyond age and gender were not collected for the control group (Supplementary Table 26).

Laboratory analysis (Supplementary Table 2) revealed that ICU-S patients had a significantly lower median white blood cell count ((WBC): 9.5 × 10³/ml vs. 13.2 × 10³/ml in ICU-NS, *p* = 0.02). Platelet counts were significantly higher in ICU-S (274.5 × 10⁶/ml) than in ICU-NS (212.7 × 10⁶/ml), with a p-value of 0.02. ICU-S patients also exhibited lower levels of C-reactive protein (CRP) and D-dimer, with median values of 56.5 mg/L and 0.98 mg/ml, respectively, compared to 108.06 mg/L and 2.4 mg/ml in ICU-NS, both achieving statistical significance (p-values of 0.008 and 0.004, respectively). Additionally, ICU-S patients had higher median albumin levels (2.75 g/dl) compared to ICU-NS (2.4 g/dl), with a p-value of 0.02, and lower median ferritin levels (797 ng/ml compared to 1185 ng/ml in ICU-NS), with a p-value of 0.04. Other laboratory parameters, including lymphocyte and monocyte counts, INR, IL−6, hemoglobin, ALT, AST, and creatinine, did not differ significantly between groups. Detailed demographics and laboratory findings for each technique is summarized in the supplementary materials.

### Disrupted neutrophil homeostasis in severe patients

**Fig. 1:**
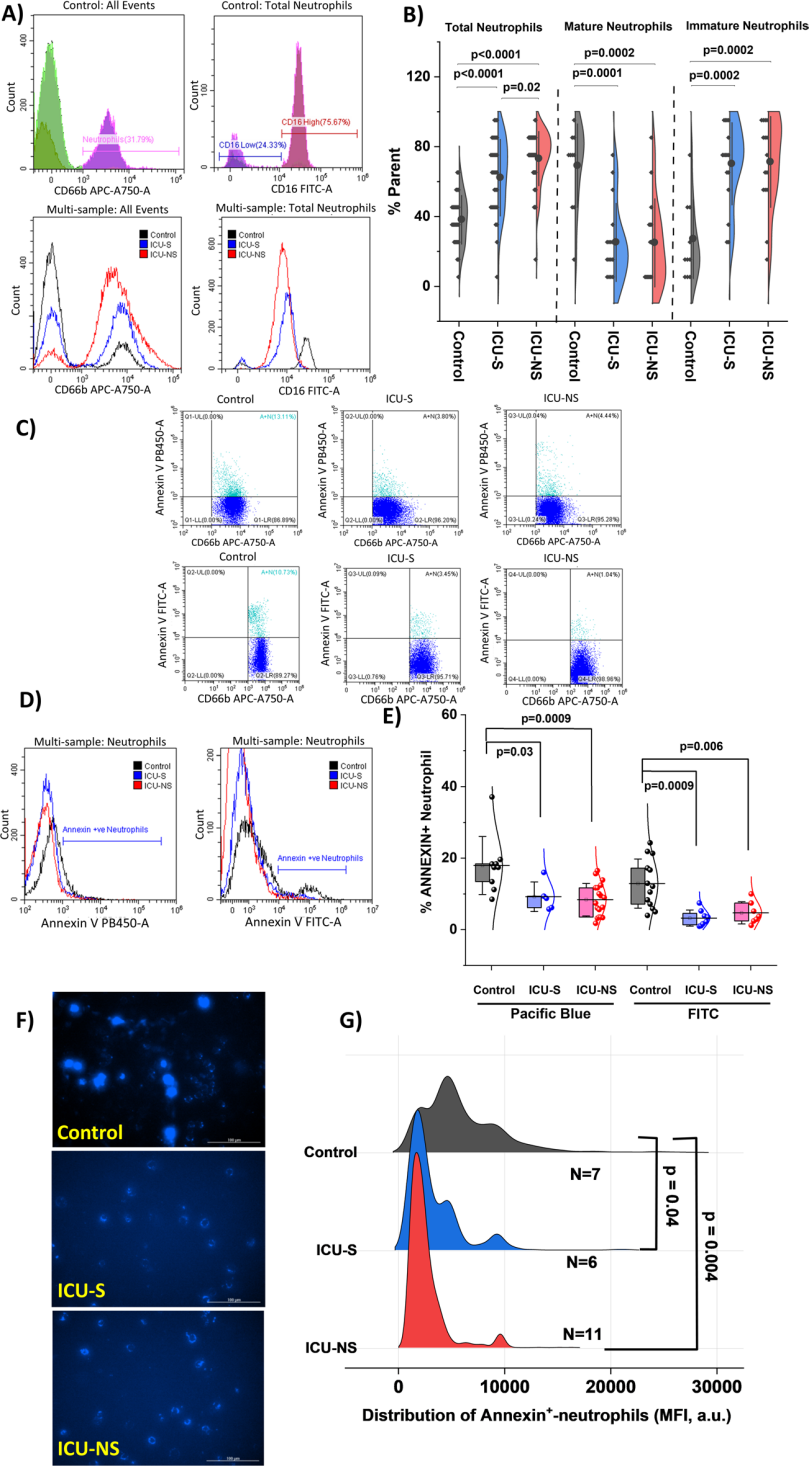
Increased neutrophil counts and dysregulated homeostasis in severe patients. (**A**) Representative flow-cytometry plots showing the gating strategy to identify total neutrophils and mature (CD16hi) versus immature (CD16low) subsets in healthy controls, ICU-S, and ICU-NS. Single-color controls and compensation matrices were applied; doublets/debris were excluded prior to analysis. (**B**) Half-violin plots of blood neutrophil proportion with overlaid individual data points and mean ± SD (controls *n* = 27, ICU-S *n* = 32, ICU-NS *n* = 48), alongside the shift toward immature neutrophils (controls *n* = 10, ICU-S *n* = 16, ICU-NS *n* = 15). (**C**, **D**) Representative annexin V staining by flow cytometry using FITC and Pacific Blue conjugates to assess early apoptosis. (**E**) Quantification of annexin V positive neutrophils (Pacific Blue: controls *n* = 9, ICU-S *n* = 5, ICU-NS *n* = 18; FITC: controls *n* = 13, ICU-S *n* = 8, ICU-NS *n* = 7). (**F**) Fluorescence images of annexin V in freshly isolated neutrophils (Cytation 5 Cell Imaging Multi-Mode Reader; identical exposure/gain settings across groups). (**G**) Per-cell annexin V intensity distributions (controls; *n* = 1606; *N* = 7 donors, ICU-S; *n* = 695; *N* = 6, ICU-NS; *n* = 1433; *N* = 11). Scale bar, 100 μm. 10,000 events were acquired and recorded for each flow cytometry sample. Exact p values are shown in panels. Data are shown as mean ± SD for violin plots and per-cell distributions. ANOVA was done followed by Tukey’s post-hoc tests to compare the differences between the three groups.

To investigate changes associated with severe COVID−19 pathologies, we analyzed neutrophil counts, phenotypes, and apoptotic profiles. We first used flow cytometry to determine the percentage of neutrophils in all patients (Fig. 1A,B). Consistent with our previous findings^57^, neutrophil counts across the Control (38.1%±13.9%; *n* = 27), ICU-S (62.3%±21.9%; *n* = 32), and ICU-NS (73.2%±15.5%; *n* = 48) groups (ICU-S vs. Control: *p* < 0.0001; ICU-NS vs. Control: *p* < 0.0001; ICU-S vs. ICU-NS: *p* = 0.019). Neutrophilia, a common form of leukocytosis, is defined by an absolute neutrophil count exceeding two standard deviations above the control mean. The patient cohort with severe COVID−19 clearly demonstrated neutrophilia.

Neutrophil phenotypes were analyzed by flow cytometry, stratified by maturity using CD16 surface markers^66,67^. CD66b positive neutrophils were categorized as CD16high (mature neutrophils) or CD16low (immature neutrophils) based on their CD16 mean fluorescence intensity (MFI) (Fig. 1A). We observed a significant decrease in CD16 high neutrophils (% parent) (control: 69.2%±23.9%; *n* = 10; ICU-S: 25.2%±22.4%; *n* = 16; ICU-NS: 24.9%±25.2%; *n* = 15; ICU-S vs. control: *p* = 0.0001; ICU-NS vs. control: *p* = 0.0002; ICU-S vs. ICU-NS: *p* = 1) and an increase in CD16 low neutrophils (% parent) (control: 27.1%±22.6%; *n* = 10; ICU-S: 70.2%±23.5%; *n* = 16; ICU-NS: 71.3%±26.0%; *n* = 15; ICU-S vs. control: *p* = 0.0002; ICU-NS vs. control: *p* = 0.0002; ICU-S vs. ICU-NS: *p* = 1) (Fig. 1B). The MFI of CD16 was also significantly lower in ICU patients compared to healthy controls (Supplementary Fig. 1). These findings are consistent with reports that immature neutrophils are rapidly mobilized from the bone marrow during severe COVID−19^9,68^. The enrichment of immature (CD16low) neutrophils is a pathophysiologic hallmark of severe COVID−19 (emergency granulopoiesis) and thus part of the phenotype under study, not a confounder to be excluded.

To investigate additional aspects of neutrophil accumulation, we assessed apoptosis in freshly isolated neutrophils from all groups by measuring the percentage of annexin-V+ cells using flow cytometry (Fig. 1C–E) and fluorescence imaging (Fig. 1F,G). Our flow cytometry analysis revealed that neutrophils from both COVID−19 groups exhibited significantly reduced annexin-V+ populations (early apoptosis) compared to the control group when using 2 different Annexin V fluorochromes (Pacific Blue: Control: 17.95% ± 8.11%; *n* = 9, ICU-S: 9.21% ± 4.11%; *n* = 5, ICU-NS: 8.40% ± 4.56%; *n* = 18. ICU-S vs. control: *p* = 0.03; ICU-NS vs. Control: *p* = 0.0009; ICU-S vs. ICU-NS: *p* = 0.96; FITC: Control: 12.88% ± 6.86%; *n* = 13, ICU-S: 3.23% ± 2.26%; *n* = 8, ICU-NS: 4.70% ± 3.09%; *n* = 7. ICU-S vs. control: *p* = 0.0009; ICU-NS vs. Control: *p* = 0.006; ICU-S vs. ICU-NS: *p* = 0.84). These results were corroborated by the quantification of MFI of individual neutrophil fluorescence images (1–5 images per subject) from controls (*N* = 7), ICU-S (*N* = 6), and ICU-NS (*N* = 11) stained with annexin V dye (Fig. 1F,G). These data suggests that the suppression of neutrophil apoptosis/clearance associates with neutrophilia in critically ill COVID−19 patients compared to healthy controls (Control: 6039 ± 2351; *N* = 7, ICU-S: 3421 ± 1773; *N* = 6, ICU-NS: 2782 ± 1440; *N* = 11. ICU-S vs. control: *p* = 0.04; ICU-NS vs. control: *p* = 0.004; ICU-S vs. ICU-NS: *p* = 0.77).

### Exploratory miRNA profiling and functional apoptosis assessment in neutrophils

**Fig. 2:**
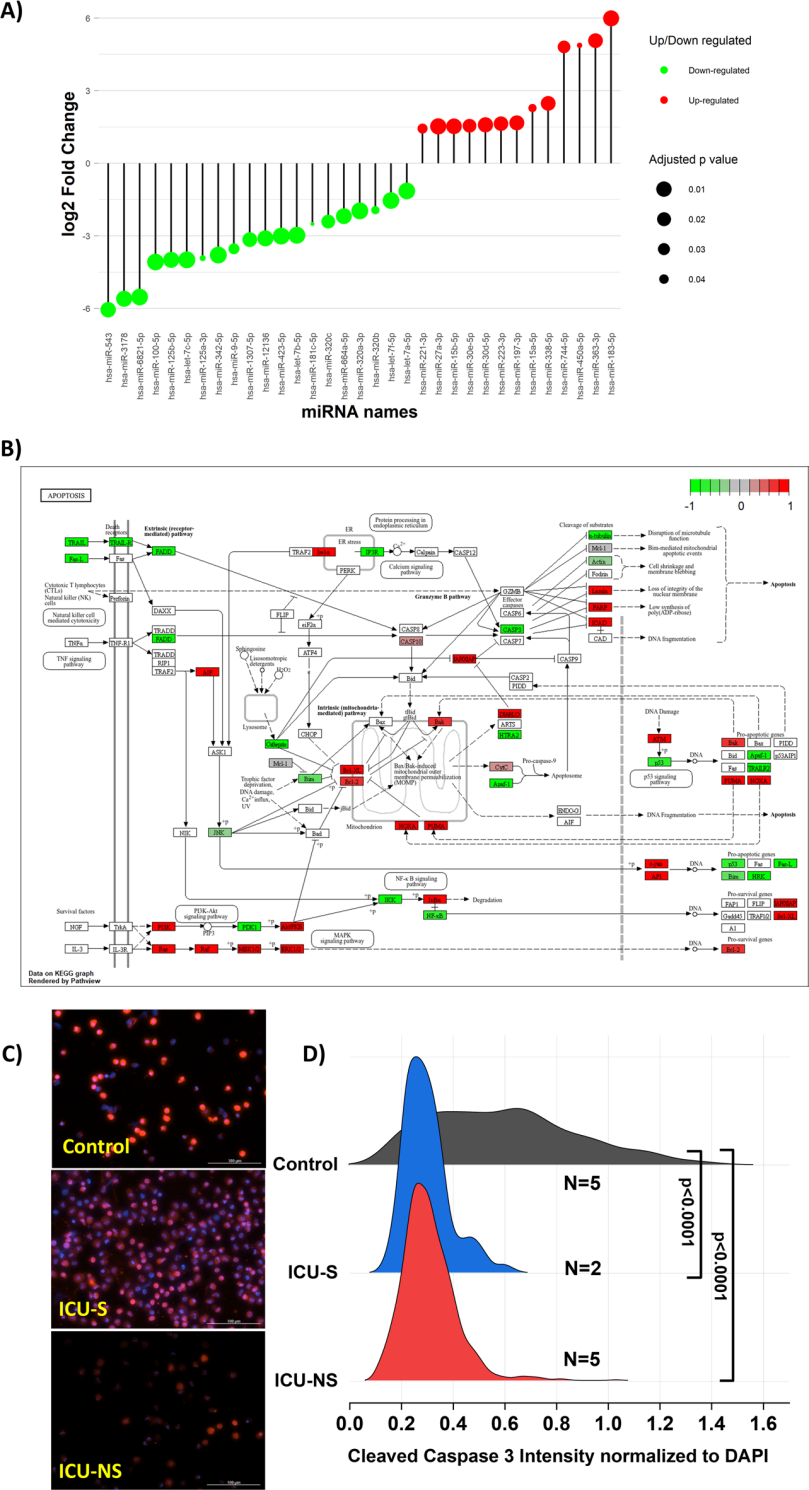
Differentially expressed neutrophil miRNAs and impaired apoptotic activation (**A**) A lollipop plot represents differentially expressed miRNA of non-survivors (*n* = 4) relative to survivors (*n* = 2) purified neutrophils. The x-axis represents miRNA ID, and the y-axis represents log2 fold changes. Each lollipop represents a differentially expressed miRNA between the two groups, with green lollipops indicating upregulated miRNAs and red lollipops indicating downregulated miRNAs. The size of the dot represents the significance level of the change in gene expression. (**B**) Apoptosis pathway map (hsa04210) rendered with Pathview with differential expression overlaid. Pathway map obtained from the Kyoto Encyclopedia of Genes and Genomes^61,63^. Copyright Kanehisa Laboratories. Reproduced with permission. KEGG apoptosis pathway visualization built from predicted miRNA–target relationships (miRCarta; experimentally supported targets only). Boxes denote genes; fill follows the color scale in the panel to indicate predicted direction/magnitude based on miRNA regulation (note: this diagram reflects predicted targets from miRNA data and is not direct mRNA/protein measurement). (**C**) Representative cleaved caspase−3 (CC3) immunofluorescence in freshly isolated neutrophils (uniform acquisition parameters). (**D**) Per-cell CC3 intensity normalized to DAPI (controls; n: 627 cells, *N* = 5; ICU-S; n: 461 cells, *N* = 2; ICU-NS; n: 1,178 cells, *N* = 5). Scale bar, 100 μm. CC3 intensity was reduced in both ICU-S and ICU-NS compared with controls, but did not differ between ICU-S and ICU-NS (*p* = 0.62). Data are mean ± SD. ANOVA was done followed by Tukey’s post-hoc tests to compare the differences between the three groups.

Thirteen miRNAs were significantly upregulated, and 20 miRNAs were significantly downregulated (adj *p* < 0.05) in ICU-NS compared with ICU-S, as shown in Fig. 2A. In this study we performed miRNA sequencing only (not bulk RNA-seq or proteomics); consequently, pathway inferences reflect miRNA-mediated regulation of target genes and are exploratory. miRNAs regulate gene expression post-transcriptionally by binding to target mRNAs and either blocking translation or promoting mRNA degradation; increased miRNA expression is generally expected to suppress corresponding target genes, and decreased miRNA expression may permit higher target expression^69–72^. Based on these established principles, we used predicted and experimentally supported miRNA–target interactions to perform pathway enrichment analysis. This analysis highlighted apoptosis- and calcium-related pathways among the putative targets of differentially expressed miRNAs (Fig. 2B), but we did not directly measure target mRNA or protein levels and therefore cannot confirm these regulatory relationships.

To functionally assess apoptosis, we performed cleaved caspase−3 (CC3) immunofluorescence imaging in freshly isolated neutrophils. CC3 intensity (normalized to DAPI) was reduced in ICU-S and ICU-NS neutrophils compared with healthy controls (Control: 0.60 ± 0.28, *n* = 627 cells from *N* = 5; ICU-S: 0.30 ± 0.09, *n* = 461 cells from *N* = 2; ICU-NS: 0.31 ± 0.10, *n* = 1,178 cells from *N* = 5; ICU-S vs. control, *p* < 0.0001; ICU-NS vs. control, *p* < 0.0001). However, CC3 intensity did not differ between ICU-S and ICU-NS (*p* = 0.62; Fig. 2C,D). Thus, while reduced CC3 in ICU patients versus controls supports impaired neutrophil apoptosis in severe COVID−19, the functional apoptosis readouts do not validate a distinct mortality-associated apoptosis program predicted by the miRNA signatures. The miRNA-based mortality signal should therefore be interpreted as hypothesis-generating and not as direct evidence of impaired apoptotic clearance in non-survivors.

### Altered calcium dynamics with decreased intracellular calcium in neutrophils from COVID−19 patients

**Fig. 3:**
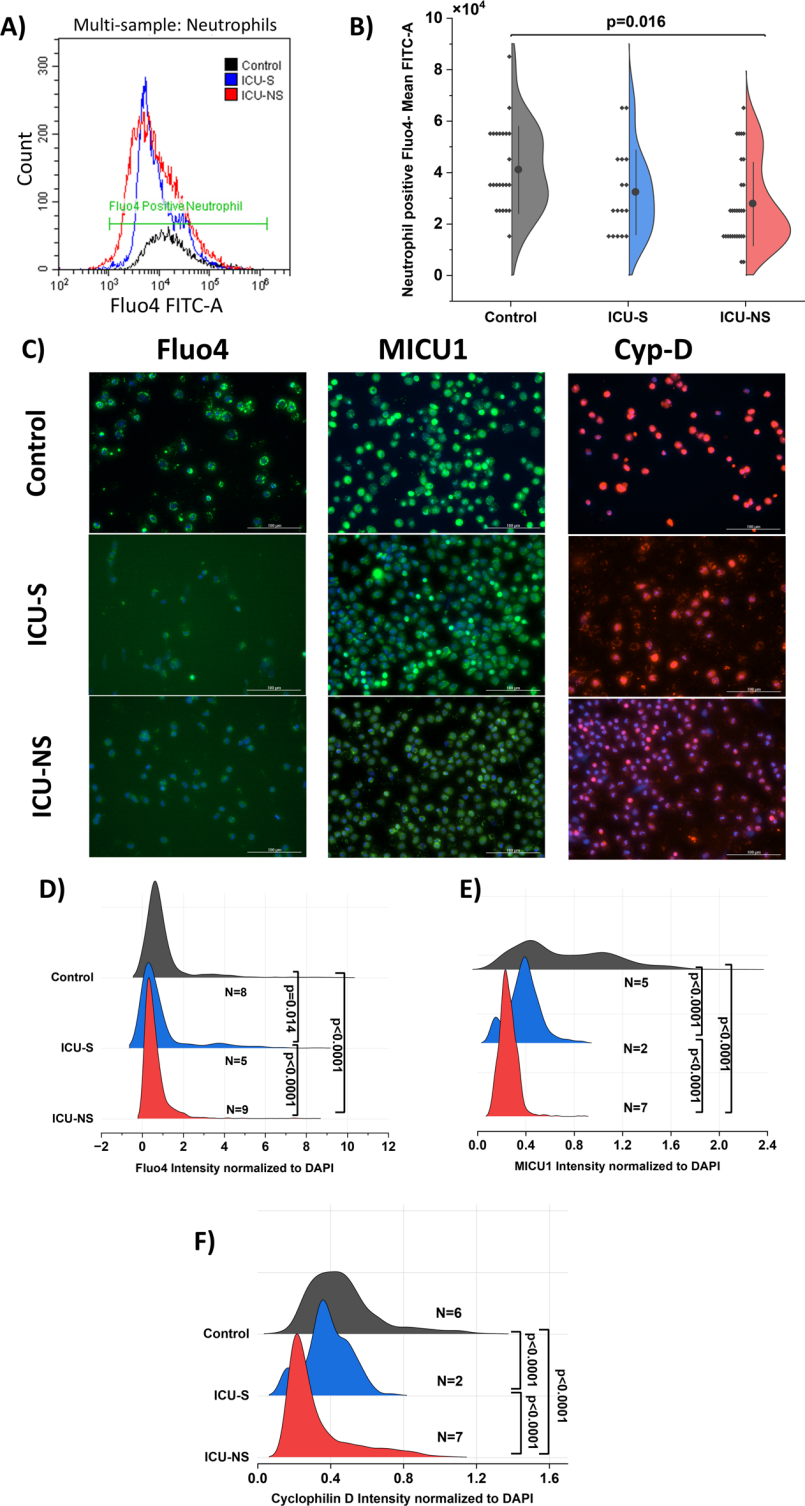
Altered calcium dynamics in COVID−19 neutrophils: reduced cytosolic Ca²⁺ and Ca²⁺-regulatory proteins ((**A**) Representative flow-cytometry histograms of Fluo−4 (cytosolic Ca²⁺ indicator) in CD66b+ neutrophils. (**B**) Half-violin plots of Fluo−4 mean fluorescence intensity with individual data points and mean ± SD (controls *n* = 23, ICU-S *n* = 16, ICU-NS *n* = 28). (**C**) Immunofluorescence for Fluo−4, MICU1 (mitochondrial Ca²⁺ uptake gatekeeper), and cyclophilin D (CypD; regulator of the mitochondrial permeability transition pore, mPTP) with DAPI nuclear counterstain. (**D**–**F**) Per-cell intensity distributions (normalized to DAPI to reduce acquisition/adherence variability): Fluo−4 (controls: 2,773 cells, *N* = 8; ICU-S: 659 cells, *N* = 5; ICU-NS: 1,668 cells, *N* = 9), MICU1 (controls: 976, *N* = 5; ICU-S: 449, *N* = 2; ICU-NS: 1,337, *N* = 7), and CypD (controls: 1,109, *N* = 6; ICU-S: 314, *N* = 2; ICU-NS: 2,075, *N* = 7). Images were acquired on a Cytation 5 with identical settings across groups; apparent DAPI differences reflect biological variation. Scale bar, 100 μm. 10,000 events were acquired and recorded for each flow cytometry sample. Data are mean ± SD. ANOVA was done followed by Tukey’s post-hoc tests to compare the differences between the three groups.

To explore the cellular factors contributing to delayed apoptosis, we studied two key regulators: calcium dynamics, and mitochondria^73,74^. We explored changes in calcium levels using flow cytometry where CD66b positive neutrophil populations were analyzed using Fluo4 dye staining to determine intracellular calcium levels (Fig. 3A). Mean fluorescence intensity of Fluo4 in neutrophils was significantly lower in severe patients, indicating reduced intracellular calcium compared to healthy controls (control: 4.1 × 10^4^± 1.7 × 10^4^; *n* = 23, ICU-S: 3.2 × 10^4^ ± 1.6 × 10^4^; *n* = 16, ICU-NS: 2.8 × 10^4^ ± 1.6 × 10^4^; *n* = 28. ICU-S vs. control: *p* = 0.25; ICU-NS vs. control: *p* = 0.016; ICU-S vs. ICU-NS: *p* = 0.66; Fig. 3B). To confirm this result and understand more regarding molecular calcium machinery inside neutrophils’ mitochondria, fluorescence imaging for Fluo4, MICU1 (mitochondrial calcium uptake regulator) and cyclophilin D (CypD, a regulator of mitochondrial permeability transition pore (mPTP)) was performed on freshly isolated neutrophils from all groups along with Hoechst 33,342 for nuclei (Fig. 3C). Fluorescent images were analyzed as described, using the MFI of Fluo4/ CypD/ MICU1 normalized to DAPI to control for dye uptake variability. Results confirmed reduced intracellular calcium (Control: 1.0 ± 1.2; *n* = 2773 cells from *N* = 8 subjects, ICU-S: 0.88 ± 1.3; *n* = 659 cells from *N* = 5 subjects, ICU-NS: 0.63 ± 0.7; *n* = 1668 cells from *N* = 9 subjects. ICU-S vs. control: *p* = 0.014; ICU-NS vs. control: *p* < 0.0001; ICU-S vs. ICU-NS: *p* < 0.0001, Fig. 3D) accompanied by decreased levels of the calcium regulators, MICU1 (Control: 0.72 ± 0.4; *n* = 976 cells from *N* = 5 subjects, ICU-S: 0.38 ± 0.13; *n* = 449 cells from *N* = 2 subjects, ICU-NS: 0.25 ± 0.07; *n* = 1337 cells from *N* = 7 subjects. ICU-S vs. Control: *p* < 0.0001; ICU-NS vs. Control: *p* < 0.0001; ICU-S vs. ICU-NS: *p* < 0.0001, Fig. 3E) and cyclophilin D (Control: 0.47 ± 0.19; *n* = 1109 cells from *N* = 6 subjects, ICU-S: 0.38 ± 0.12; *n* = 314 cells from *N* = 2 subjects, ICU-NS: 0.32 ± 0.18; *n* = 2075 cells from *N* = 7 subjects. ICU-S vs. control: *p* < 0.0001; ICU-NS vs. control: *p* < 0.0001; ICU-S vs. ICU-NS: *p* < 0.0001, Fig. 3F).

### Mitochondria from neutrophils of severe COVID−19 patients exhibit hyperpolarized transmembrane potential and increased oxygen consumption

**Fig. 4:**
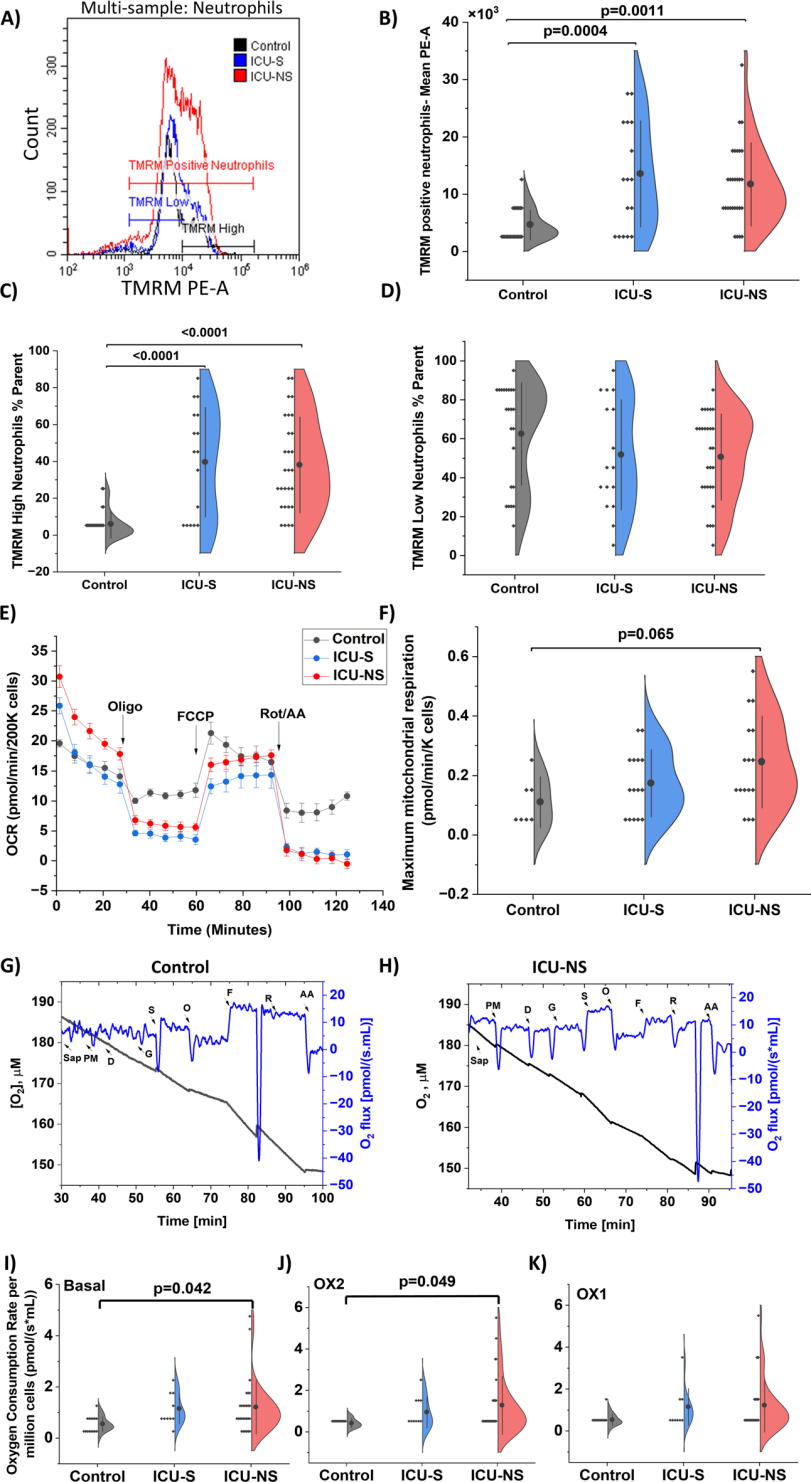
Mitochondria from neutrophils of severe COVID−19 patients exhibit hyperpolarized transmembrane potential and increased oxygen consumption. (**A**) Representative histograms for flow cytometry comparing TMRM positive neutrophils in control, ICU-S and ICU-NS subjects. (**B**) A half violin plot showing a significant increase in transmembrane potential of neutrophils in COVID-ICU patients compared to control group (*N* = 23, 16 and 28 for control, ICU-S and, ICU-NS; respectively). (**C**) A half violin plot showing a significant increase in percentage of neutrophil high TMRM population in COVID-ICU patients compared to control group (*N* = 22, 16 and 28 for control, ICU-S and, ICU-NS; respectively). (**D**) A half violin plot showing a non-significant change in percentage of neutrophil low TMRM population in COVID-ICU patients compared to control group (*N* = 23, 16 and 28 for control, ICU-S and, ICU-NS; respectively). (**E**) Representative Seahorse XF traces of oxygen consumption rate (OCR) in freshly isolated neutrophils (key injections annotated). (**F**) A half violin plot showing a trend of increase in maximal mitochondrial respiration in neutrophils from ICU-NS patients compared to controls (*N* = 7, 15 and 16 for control, ICU-S and, ICU-NS; respectively); 2 × 10^5^ neutrophils/well. (**G**, **H**) Representative traces of high resolution respirometry showing OCR of freshly isolated neutrophils from control and ICU-NS group. (I-K) Half violin plots showing OCR per million cells. (**I**) A half violin plot showing a significant increase in OCR at basal state between control and ICU-NS groups (*n* = 15, 10 and 28 for control, ICU-S and, ICU-NS; respectively). (**J**) A half violin plot showing a significant increase in OCR upon complex II stimulation between control and ICU-NS groups (*N* = 14, 10 and 28 for control, ICU-S and, ICU-NS; respectively). (**K**) A half violin plot showing non-significant change in OCR upon complex I stimulation between control and ICU-patients (*N* = 14, 9 and 28 for control, ICU-S and, ICU-NS; respectively). 10,000 events were acquired and recorded for each flow cytometry sample. All data presented as mean ± SD. ANOVA was done followed by Tukey’s post-hoc tests to compare the differences between the three groups.

Given calcium’s role in apoptosis regulation, we investigated its role in mitochondrial energy production. First, we investigated the mitochondrial dynamics and their changes in response to decreased intracellular calcium levels. TMRM staining was performed on CD66b positive neutrophil populations to assess mitochondrial transmembrane potential ∆Ψ_m_ (Fig. 4A). Distribution of TMRM means fluorescence intensity indicates hyperpolarized mitochondria in both ICU-S and ICU-NS groups (Control: 4.6 × 10^3^± 2.6 × 10^3^; *n* = 23, ICU-S: 13.5 × 10^3^ ± 9.3 × 10^3^; *n* = 16, ICU-NS: 11.7 × 10^3^ ± 7.3 × 10^3^; *n* = 28. ICU-S vs. control: *p* = 0.0004; ICU-NS vs. control: *p* = 0.0011; ICU-S vs. ICU-NS: *p* = 0.66, Fig. 4B). Moreover, we stratified the TMRM neutrophil positive population into ‘TMRM-high’ and ‘TMRM-low’ as shown in panel (A). Our analysis showed a significant increase in the mean TMRM MFI associated with the mitochondria of freshly isolated neutrophils from patients with severe disease compared to healthy controls. This increase in TMRM uptake was concomitantly associated with increased TMRM-high (control: 5.8%± 7.4%; *n* = 22, ICU-S: 39.5% ± 29.8%; *n* = 16, ICU-NS: 38.0% ± 26%; *n* = 28). ICU-S vs. control: *p* < 0.0001; ICU-NS vs. control: *p* < 0.0001; ICU-S vs. ICU-NS: *p* = 0.97, Fig. 4C), but not TMRM-low neutrophil populations in both ICU-S and ICU-NS groups (control: 62.5%± 26.3%; *n* = 23, ICU-S: 51.8% ± 28.3%; *n* = 16, ICU-NS: 50.6% ± 22.2%; *n* = 28. ICU-S vs. control: *p* = 0.40; ICU-NS vs. control: *p* = 0.22; ICU-S vs. ICU-NS: *p* = 0.99, Fig. 4D), suggesting that neutrophilia in severe COVID−19 is associated with hyperpolarized mitochondria.

We then explored whether these changes in dynamics reflected mitochondrial respiratory function in neutrophils freshly isolated from a cohort of ICU-hospitalized subjects, using a seahorse XF analyzer and high-resolution respirometry (Fig. 4E–K). Maximal mitochondrial respiration, calculated as the difference between FCCP and Rotenone/Antimycin A states, tended to increase in ICU-NS neutrophils compared to controls (Control: 0.11 ± 0.09; *n* = 7, ICU-S: 0.17 ± 0.11; *n* = 15, ICU-NS: 0.25 ± 0.15; *n* = 16. ICU-S vs. control: *p* = 0.53; ICU-NS vs. control: *p* = 0.065; ICU-S vs. ICU-NS: *p* = 0.28, Fig. 4F). Furthermore, mitochondrial respiration of the ICU-NS neutrophils was significantly increased at the basal level (Control: 0.55 ± 0.30; *n* = 15, ICU-S: 1.15 ± 0.60; *n* = 10, ICU-NS: 1.21 ± 1.04; *n* = 28. ICU-S vs. Control: *p* = 0.19; ICU-NS vs. Control: *p* = 0.042; ICU-S vs. ICU-NS: *p* = 0.98, Fig. 4I) or upon complex II stimulation (Control: 0.41 ± 0.22; *n* = 14, ICU-S: 0.947 ± 0.76; *n* = 10, ICU-NS: 1.28 ± 1.4; *n* = 28. ICU-S vs. Control: *p* = 0.47; ICU-NS vs. Control: *p* = 0.049; ICU-S vs. ICU-NS: *p* = 0.69, Fig. 4J) and showed a non-significant increase upon complex I stimulation (Control: 0.53 ± 0.28; *n* = 14, ICU-S: 1.15 ± 0.88; *n* = 9, ICU-NS: 1.22 ± 1.28; *n* = 28. ICU-S vs. control: *p* = 0.35; ICU-NS vs. control: *p* = 0.11; ICU-S vs. ICU-NS: *p* = 0.98, Fig. 4K), indicating an increased oxidative metabolism to support immune cell activation in ICU-NS patients. These changes in mitochondrial respiration were supported by miRNA data, suggesting an increase in complexes 1, 2, and 4 and a decrease in complex 3 (Supplementary Table 3).

### Mitochondria from non-survivors show fragmented phenotype and increased ROS production

**Fig. 5:**
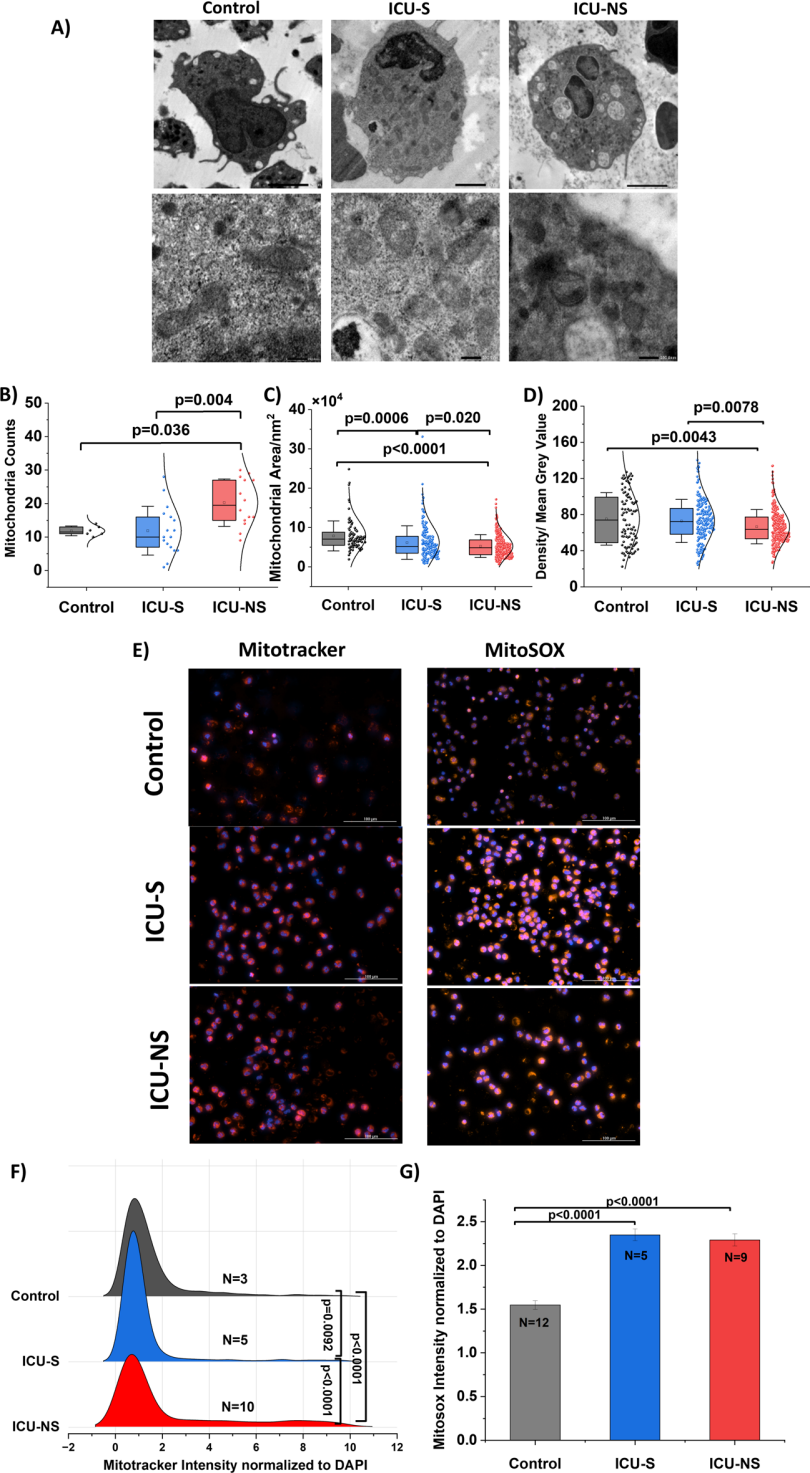
Mitochondria from non-survivors show fragmented phenotype and increased ROS production. (**A**) Representative electron microscope images showing mitochondrial distribution (upper panel, scale bar 2 μm) and mitochondrial characteristics (lower panel, 200, 500 nm). (**B**) A half violin plot showing a significant increase in mitochondria per neutrophil from ICU-S and ICU-NS patients compared to control (*N* = 4, 5 and, 4 for control, ICU-S and, ICU-NS; respectively). (**C**, **D**) A half violin plot showing a significant decrease in mitochondria area and mean grey value indicating mitochondria density (*n* = 91, 168 and, 290 for control, ICU-S and, ICU-NS; respectively). All data presented as mean ± SD. (**E**) Representative fluorescence images for MitoTracker (mitochondrial content) and MitoSOX (mitochondrial superoxide) with DAPI; images acquired on Cytation 5 (objective 20×) and analysis was performed using Gen5 Software. Scale bar: 100 μm. (**F**) MitoTracker per-cell intensity normalized to DAPI (controls; n: 1,129 cells, *N* = 3; ICU-S; n: 1,358 cells, *N* = 5; ICU-NS; n: 2,366 cells, *N* = 10); half-violin with individual points and mean ± SD. (**G**) MitoSOX per-cell intensity normalized to DAPI (controls; n: 4026 cells, *N* = 12; ICU-S; n: 2,615 cells, *N* = 5; ICU-NS; n: 3,022 cells, *N* = 9) displayed as a bar graph (mean with SEM); exact p values in panel. MitoSOX intensity was increased in ICU-S and ICU-NS relative to controls but did not differ significantly between ICU-S and ICU-NS (*p* = 0.8). ANOVA was done followed by Tukey’s post-hoc tests to compare the differences between the three groups.

To further examine changes in mitochondria, we analyzed Transmission Electron Microscopy (TEM) images to assess mitochondrial counts and morphology of representative neutrophils from the control (*n* = 4), ICU-S (*n* = 5), and ICU-NS (*n* = 4) groups (Fig. 5A–D). Neutrophils from ICU-NS showed significantly higher mitochondrial counts per cell (Control: 11.8 ± 1.5, ICU-S: 11.8 ± 7.3, ICU-NS: 20.3 ± 7.1; (*n* = 6–17 neutrophils). ICU-S vs. control: *p* = 1; ICU-NS vs. control: *p* = 0.036; ICU-S vs. ICU-NS: *p* = 0.004, Fig. 5B). Interestingly those mitochondria showed decreased mean cross-sectional area (Control: 7.9 × 10^4^ ± 3.8 × 10^4^; *n* = 91 mitochondria, ICU-S: 6.2 × 10^4^ ± 4.3 × 10^4^; *n* = 186 mitochondria, ICU-NS: 5.3 × 10^4^ ± 2.9 × 10^4^; *n* = 290 mitochondria. ICU-S vs. Control: *p* = 0.0006; ICU-NS vs. Control: *p* < 0.0001; ICU-S vs. ICU-NS: *p* = 0.02, Fig. 5C) and decreased mean grey value reflecting mitochondrial density (Control: 75.2 ± 29.1; *n* = 91 mitochondria, ICU-S: 73.0 ± 23.8; *n* = 186 mitochondria, ICU-NS: 66.6 ± 19.0; *n* = 290 mitochondria. ICU-S vs. control: *p* = 0.711; ICU-NS vs. control: *p* = 0.0043; ICU-S vs. ICU-NS: *p* = 0.0078, Fig. 5D). These data indicate that neutrophil mitochondria in COVID−19 patients are small and fragmented, reflecting a stressed phenotype.

To confirm the changes in mitochondrial density, MitoTracker staining of isolated neutrophils showed an increase in MitoTracker fluorescence intensity normalized to DAPI in ICU COVID−19 patients compared to controls (Control: 1.44 ± 1.4; *n* = 1129 cells from *N* = 3 subjects, ICU-S: 1.20 ± 1.4; *n* = 1358 cells from *N* = 5 subjects, ICU-NS: 2.18 ± 2.6; *n* = 2366 cells from *N* = 10 subjects. ICU-S vs. control: *p* = 0.0092; ICU-NS vs. control: *p* < 0.0001; ICU-S vs. ICU-NS: *p* < 0.0001, Fig. 5E and F). This mitochondrial phenotype suggests ROS-producing mitochondria; therefore, mitochondrial ROS levels were assessed using MitoSOX dye normalized to DAPI. ICU COVID−19 neutrophils exhibited elevated mitochondrial ROS compared to controls (Control: 1.55 ± 3.1; *n* = 4026 cells from *N* = 12 subjects, ICU-S: 2.35 ± 3.5; *n* = 2615 cells from *N* = 5 subjects, ICU-NS: 2.30 ± 3.9; *n* = 3022 cells from *N* = 9 subjects. ICU-S vs. control: *p* < 0.0001; ICU-NS vs. control: *p* < 0.0001; ICU-S vs. ICU-NS: *p* = 0.8, Fig. 5E,G).

## Discussion

Severe COVID−19 in our cohort was characterized by circulating neutrophilia with an immature shift, reduced apoptosis, hyperpolarized mitochondria, and elevated mitochondrial ROS relative to healthy controls. Building on prior descriptions of emergency granulopoiesis in COVID−19, we sought to characterize the total circulating neutrophil phenotype as it exists in patients and to identify mortality-associated differences within ICU cohorts. Relative to healthy controls, COVID−19 patients showed neutrophilia with increased CD16low fractions, reduced apoptosis (annexin V, cleaved caspase−3), lower cytosolic Ca²⁺, reduced MICU1 and cyclophilin D, hyperpolarized ΔΨm, and increased mROS. These observations are consistent with impaired apoptotic clearance and mitochondrial activation within an immature-enriched circulating pool but do not establish causality. Within ICU cohorts, where maturity proportions were similar across experimental subsets, non-survivors exhibited higher basal and complex II–driven respiration and stronger ΔΨm shifts; exploratory miRNA profiling suggested tighter regulation of apoptosis-related pathways in non-survivors. However, functional apoptosis assays (annexin V and cleaved caspase−3) did not differ between ICU-S and ICU-NS, indicating that mortality-linked miRNA changes should be viewed as hypothesis-generating rather than as validated determinants of outcome.

### Disrupted neutrophil homeostasis and apoptosis

This study contributes to the growing body of literature documenting increased neutrophil counts in severe COVID−19 cases^57,75–80^. We found that a higher population of CD16low neutrophils indicate immature neutrophils, with a corresponding decrease in CD16high, mature neutrophils in severely ill patients. Prior studies have linked immature neutrophils to infections^81,82^ and the immature phenotype in COVID−19 has been attributed to emergency myelopoiesis and dysfunctional mature neutrophils^8,9,83^.

Concurrently, circulating neutrophils were significantly less apoptotic in critically ill patients compared with healthy controls. However, apoptosis markers did not differ between ICU-S and ICU-NS. Multiple groups have now reported altered circulating and cellular miRNA profiles in COVID−19 and long COVID, with signatures linked to inflammation, thrombosis, and organ injury^84–88^. Our miRNA-seq analysis should therefore be viewed as an exploratory contribution to this emerging miRNA landscape. Our miRNA profiling indicates regulatory signatures that could be consistent with down-modulation of apoptotic pathways in non-survivors, but we did not directly quantify target mRNA or protein levels, and these regulatory inferences therefore require validation. Mechanistically, the intrinsic (BCL−2 family – cytochrome c/Apaf−1/caspase−9 – caspase−3) and extrinsic (death receptor–mediated) apoptosis pathways converge on the mitochondrion and are compatible with our Ca²⁺–mitochondria framework^89^. Importantly, because functional apoptosis readouts (annexin V, cleaved caspase−3) did not distinguish ICU-S from ICU-NS, we interpret the mortality-associated miRNA signal as exploratory and do not claim a confirmed impairment of apoptotic clearance specific to non-survivors. As an independent functional assessment of apoptosis, reduced cleaved caspase−3 immunofluorescence in severe COVID−19 versus controls supports impaired apoptotic clearance and disrupted neutrophil homeostasis, in line with previous observations in tracheal aspirate neutrophils^90^. This impaired apoptosis may contribute to neutrophil accumulation and could worsen the histotoxic-destructive capacity of neutrophils in tissues and organs, although we did not directly assess tissue injury in this cohort^91^.

### Alternative interpretations and systemic confounders

Several alternative explanations could account for the neutrophil phenotypes we describe. First, the observed mitochondrial hyperpolarization, increased complex II–linked respiration, and elevated mROS may reflect a generalized “viral mitochondriopathy” and systemic metabolic stress in critical illness, rather than a neutrophil-specific program that drives mortality. Similar mitochondrial dysfunction and ROS imbalance have been reported across multiple tissues and cell types in COVID−19 and long COVID (e.g., epithelial cells, lymphocytes, and endothelial cells)^39,40,92,93^. Importantly, neutrophil linked inflammatory programmes can persist beyond acute infection. Post-COVID pulmonary sequelae have been associated with a persistent neutrophil-associated immune signature and circulating markers consistent with ongoing neutrophil extracellular trap activity months after recovery^21,94,95^. This aligns with the broader concept that mitochondrial dysregulation and reactive oxygen species imbalance may contribute not only to acute severe disease but also to post-acute sequelae, providing a rationale for longitudinal studies that track neutrophil mitochondrial phenotypes from hospitalisation into convalescence^17,19^. Second, differences between ICU-S and ICU-NS may be influenced by unmeasured clinical variables such as comorbidities, timing of sampling relative to disease onset, co-infections, and exposures to drugs (for example, corticosteroids, vasopressors, and immunomodulators), which we could not fully adjust for. Third, we did not include a non-COVID critically ill control group. Thus, we cannot determine to what extent the neutrophil mitochondrial phenotype is specific to SARS-CoV−2 versus a broader response to severe systemic inflammation and hypoxia. Finally, our cross-sectional sampling design does not establish temporal ordering; mitochondrial and calcium alterations might precede clinical deterioration, occur in parallel with it, or represent a late consequence of multi-organ dysfunction. These considerations emphasize that our data identify associations and plausible mechanisms but do not confirm causality.

### Integrated Ca^2+^–mitochondria–apoptosis model

**Fig. 6:**
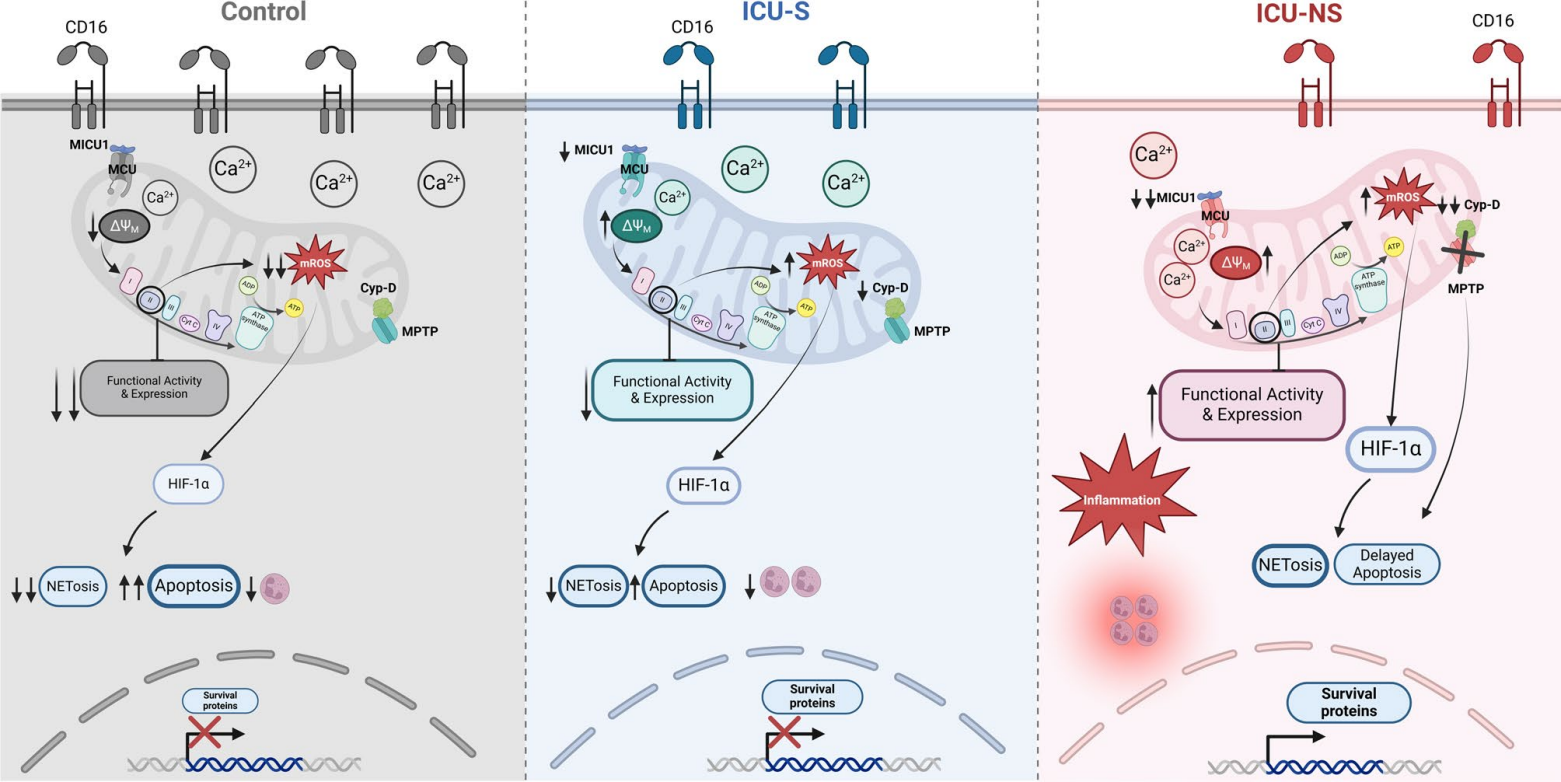
Integrated model linking Ca²⁺ handling, mitochondrial function, and neutrophil survival in severe COVID−19. Schematic summarizing study findings: reduced cytosolic Ca²⁺ (Fluo−4) and down-regulation of MICU1 and cyclophilin D (CypD) favor lower mPTP opening and, together with hyperpolarized ΔΨm, sustain mitochondrial activity and ROS generation (MitoSOX), illustrates a proposed model for neutrophils in severe COVID−19 patients. This figure was created with BioRender.com.

We reconcile the apparently paradoxical observations (lower cytosolic Ca²⁺ but sustained mitochondrial activity) as follows. (i) Cytosolic Ca²⁺ is reduced which is consistent with enhanced ER sequestration (e.g., higher SERCA activity inferred from miRNA target mapping). (ii) MICU1 reduction lowers uniporter gatekeeping, reducing maximum influx capacity. (iii) Cyclophilin D reduction limits mPTP opening, decreasing depolarization events. (iv) ΔΨm hyperpolarization preserves a strong electrochemical drive that maintains moderate mitochondrial Ca²⁺ without overload. Net effect: these findings are compatible with a model in which neutrophil apoptosis is delayed and mitochondrial respiration (notably complex II) and mROS are increased in severe COVID−19, but this model remains speculative and requires experimental testing. In our cohort, apoptosis and mROS read-outs did not differ between ICU survivors and non-survivors, so this schematic should be viewed as a conceptual framework for severe disease rather than a validated mechanism of mortality. We reflect this mechanism in the revised schematic (Fig. 6).

Calcium (Ca²⁺) is a crucial signaling molecule involved in neutrophil activation, oxidative stress, cell death/clearance, and inflammation^46,96^. Previous studies have suggested that calcium-dependent stimulation of oxidative phosphorylation (OXPHOS) occurs via specific regulatory mechanisms, although mitochondrial calcium overload can lead to deleterious inhibition of OXPHOS^97^. A decrease in cyclophilin D inCOVID−19 neutrophils^98–100^ with a decrease in MICU1 levels indicates impaired gatekeeping of the calcium uniporter and altered calcium uptake^101^. These observations were confirmed by miRNA interactions, where decreased IP3R, VDAC, cyp-D, with increased SERCA and STIM, led to enhanced calcium uptake to the Endoplasmic Reticulum (ER) with potential decreased mitochondrial calcium influx (Supplementary Table 3), indicating a triad that leads to decreased apoptotic signals^74^.

### Mitochondrial dynamics and ROS production

Although neutrophils were long considered glycolysis-dominant, accumulating evidence shows context-dependent mitochondrial contributions to chemotaxis, effector function, and cell-death programs; consistent with our observations in severe COVID−19^26,102–104^. We observed increased mitochondrial respiratory activity, particularly at complex II (succinate dehydrogenase, SDH), accompanied by a hyperpolarized mitochondrial phenotype and significant morphological changes, resulting in small, fragmented mitochondria in non-survivors. This contrasts with earlier studies reporting increased glycolytic flux^47,48^ but aligns with Reyes et al., who provided evidence of reduced glycolytic flux and a switch to oxidative metabolism in neutrophils isolated from COVID−19 patients with ARDS^49^. Since Complex II activity and mitochondrial fragmentation were previously linked to oxidative stress or ROS production^105–108^, we confirmed increased mitochondrial ROS (mROS) production in the neutrophils of patients with severe COVID−19. Elevated mROS production from complex II or GPD2, potentially triggered by hypoxia, may contribute to hypoxia-inducible factor 1-alpha (HIF−1α) stabilization by inhibiting prolyl hydroxylase domain protein 2 (PHD2)^58,109–111^. HIF signaling and the expression of PHD enzymes, particularly prolyl hydroxylase domain protein 3 (PHD3), have been implicated in the prolonged neutrophil survival under hypoxic conditions^112^. Collectively, increased mROS levels have been shown to enhance NETosis, stabilize HIF1a, and delay neutrophil apoptosis, ultimately promoting neutrophilic inflammation. Recent immunometabolic data link metabolic reprogramming to neutrophil extracellular trap formation in severe coronavirus disease 2019. Li et al. identified reduced glyceraldehyde−3-phosphate dehydrogenase activity as a key feature of severe disease and showed that perturbing this axis can promote neutrophil extracellular trap formation. In keeping with our findings of increased complex II linked respiration and mitochondrial reactive oxygen species, this supports convergence of glycolytic control points and mitochondrial redox output on neutrophil extracellular trap permissiveness, while not establishing causality^18^. Furthermore, enhanced mitochondrial respiration and ATP production facilitate chemotaxis and may contribute to neutrophil-mediated lung damage observed in severe COVID−19, although our data cannot establish a direct causal link to mortality^36,50,113^.

### Metabolic reprogramming in immature neutrophils

Previous studies reported that mature neutrophils primarily depend on glycolytic machinery, while neutrophil precursors, immature neutrophils, and low-density neutrophils rely on the mitochondrial metabolism and OXPHOS^113^. Emerging evidence challenges the traditional dogma, demonstrating that neutrophils, particularly immature phenotypes, exhibit higher global bioenergetic capacity^114,115^. These bioenergetic dynamics may explain the altered neutrophil function, such as increased chemotaxis, mROS production, and activation. Chemotaxis depends on mitochondrial metabolism; disruption of the mitochondrial membrane potential using FCCP or inhibition of mitochondrial ATP synthesis with oligomycin impairs neutrophil chemotaxis^116^. Wauters et al. identified five distinct neutrophil phenotypes in bronchoalveolar lavage fluid (BALF) and observed that “progenitor” and “inflammatory mature” neutrophils were significantly higher in COVID−19 patients^117^. Interestingly, phenotypic profiling of inflammatory immature neutrophils revealed decreased CD44 surface expression, indicating an enhanced migratory capacity towards the lungs^118,119^. This supports the notion that increased immature neutrophils in severe COVID−19, together with enhanced mitochondrial activity, may be associated with higher migratory activity and could exacerbate lung damage and adverse outcomes; however, we did not directly measure tissue infiltration or lung injury in this cohort. Our data is consistent with increased migratory activity and may be relevant to neutrophil- mediated lung injury^120^, but we could not test causality in this observational study. Additionally, increased calcium sequestration in intracellular stores, coupled with lower cytosolic calcium levels, may underlie the enhanced mitochondrial respiratory function, elevated mROS production, stabilization of pro-survival proteins, and delayed apoptosis observed in neutrophils of severe COVID−19 patients.

### Clinical implications

By locating several mortality-associated changes at the Ca2+-mitochondria interface, our data highlight MICU1-mPTP-complex II checkpoints as potential nodes of interest for future mechanistic studies of neutrophil lifespan in severe COVID−19. Potential therapeutic approaches might include agents that restore neutrophil apoptosis, mitochondrial-targeted redox modulation, or calcium channel modulators^39–42^. However, these observational associations warrant mechanistic and interventional studies to determine whether targeting these pathways can safely modify disease course, particularly given the essential role of neutrophils in host defense against secondary infections.

### Study limitations

First, we profiled miRNAs only; pathway inferences therefore reflect miRNA-mediated regulation and require mRNA/protein-level validation. In our cohort, functional apoptosis assays (annexin V and cleaved caspase−3) did not differ between ICU-S and ICU-NS, so the mortality-associated miRNA signature should be regarded as exploratory and not as validated evidence of a distinct apoptosis program in non-survivors. Second, the study is observational and single center, so associations should not be interpreted as causal. Third, the miRNA-seq sample size was modest which reduces the power for mortality comparison and full severity comparison due to the absence of healthy controls. Fourth, we analyzed the total circulating neutrophil population, which is the clinically relevant state in COVID−19 rather than sorting by maturity; survivor vs. non-survivor contrasts mitigate maturity confounding but do not eliminate it. Fifth, SOFA and APACHE II severity scores were not systematically recorded during the pandemic period, limiting our ability to adjust for baseline disease severity beyond the biomarkers reported. Sixth, no mild/moderate disease subjects were added to the study.

### Future directions

Priority next steps include paired mRNA/protein validation of miRNA-predicted nodes (e.g., MICU1, CypD, SERCA targets), functional perturbation of complex II/mPTP in ex vivo neutrophils, and prospective clinical studies to test whether apoptosis-restoring interventions can reverse the mortality-linked metabolic signature identified here.

## Conclusions

In summary, we show that neutrophils from critically ill COVID−19 patients exhibit coordinated alterations in calcium handling, mitochondrial activity, and mitochondrial morphology, and that several calcium-regulatory proteins and mitochondrial features differ between ICU survivors and non-survivors. These findings suggest that calcium–mitochondria checkpoints and mitochondrial dynamics may be characteristic of severe COVID−19 and show associations with mortality in our cohort, but these associations require confirmation in larger and independent datasets. However, this observational design does not establish causality, and we cannot determine whether these alterations are drivers or consequences of critical illness. Future mechanistic and interventional studies are needed to test whether modulating these pathways can beneficially influence neutrophil lifespan or clinical outcomes.

## Supplementary Information

Below is the link to the electronic supplementary material.

Supplementary Figure 1

Supplementary Figure 2

Supplementary Figure 3

Supplementary Figure 4

Supplementary Figure 5

Supplementary Materials

## Data Availability

All data generated or analyzed during this study are included in this article and its supplementary material files. Further inquiries can be directed to the corresponding author. The miRNA Fastq sequencing data generated in this study is available on the Sequence Read Archive (SRA) of the NCBI database under project number PRJNA1184218 while the processed files together with the codes are available on the github repository (https://github.com/Ashraf-Eltaher/Functional-and-transcriptomic-alterations-in-COVID-19-patients).

## Abbreviations

ACD: Acid citrate dextrose
ALT: Alanine aminotransferase
ARDS: Acute respiratory distress syndrome
AST: Aspartate aminotransferase
ATP: Adenosine triphosphate
BALF: Bronchoalveolar lavage fluid
BSA: Bovine serum albumin
CC3: Cleaved caspase 3
COVID-19: Coronavirus disease 2019
CRP: C-reactive protein
CypD: Cyclophilin D
DAPI: 4’,6-diamidino-2-phenylindole
DEM: Differentially expressed miRNA
DMEM: Dulbecco’s modified Eagle’s medium
ER: Endoplasmic reticulum
ETC: Electron transport chain
ETS: Electron transfer system
FCCP: Carbonyl cyanide p-(trifluoro-methoxy) phenyl-hydrazone
FiO2: Fraction of inspired oxygen
FITC: Fluorescein isothiocyanate
HBSS: Hank’s balanced salt solution
HIF-1α: Hypoxia-inducible factor 1-alpha
ICU: Intensive care unit
ICU-NS: ICU non-survivors
ICU-S: ICU survivors
IL-6: Interleukin-6
INR: International normalized ratio
MFI: Mean fluorescence intensity
miRNA: microRNA
mPTP: Mitochondrial permeability transition pore
mROS: Mitochondrial reactive oxygen species
NET: Neutrophil extracellular trap
NLR: Neutrophil-to-lymphocyte ratio
OCR: Oxygen consumption rate
OXPHOS: Oxidative phosphorylation
PBS: Phosphate-buffered saline
PFA: Paraformaldehyde
PHD2: Prolyl hydroxylase domain protein 2
PHD3: Prolyl hydroxylase domain protein 3
RBC: Red blood cell
ROS: Reactive oxygen species
RT-PCR: Reverse transcription polymerase chain reaction
SARS-CoV-2: Severe acute respiratory syndrome Coronavirus 2
SDH: Succinate dehydrogenase
sO2: Oxygen saturation
SUIT: Substrate-uncoupler-inhibitor titration
TEM: Transmission electron microscopy
TMRM: Tetramethylrhodamine methyl ester perchlorate
WBC: White blood cell
ΔΨm: Mitochondrial transmembrane potential
