# Supplementary figures and images for: Disruption of neutrophil homeostasis is associated with functional alterations in mitochondria of critically ill COVID−19 patients
Source: Sci Rep. 2026 Mar 1;16:7838. doi: 10.1038/s41598-026-38741-y (PMC12953904; doi:10.1038/s41598-026-38741-y)

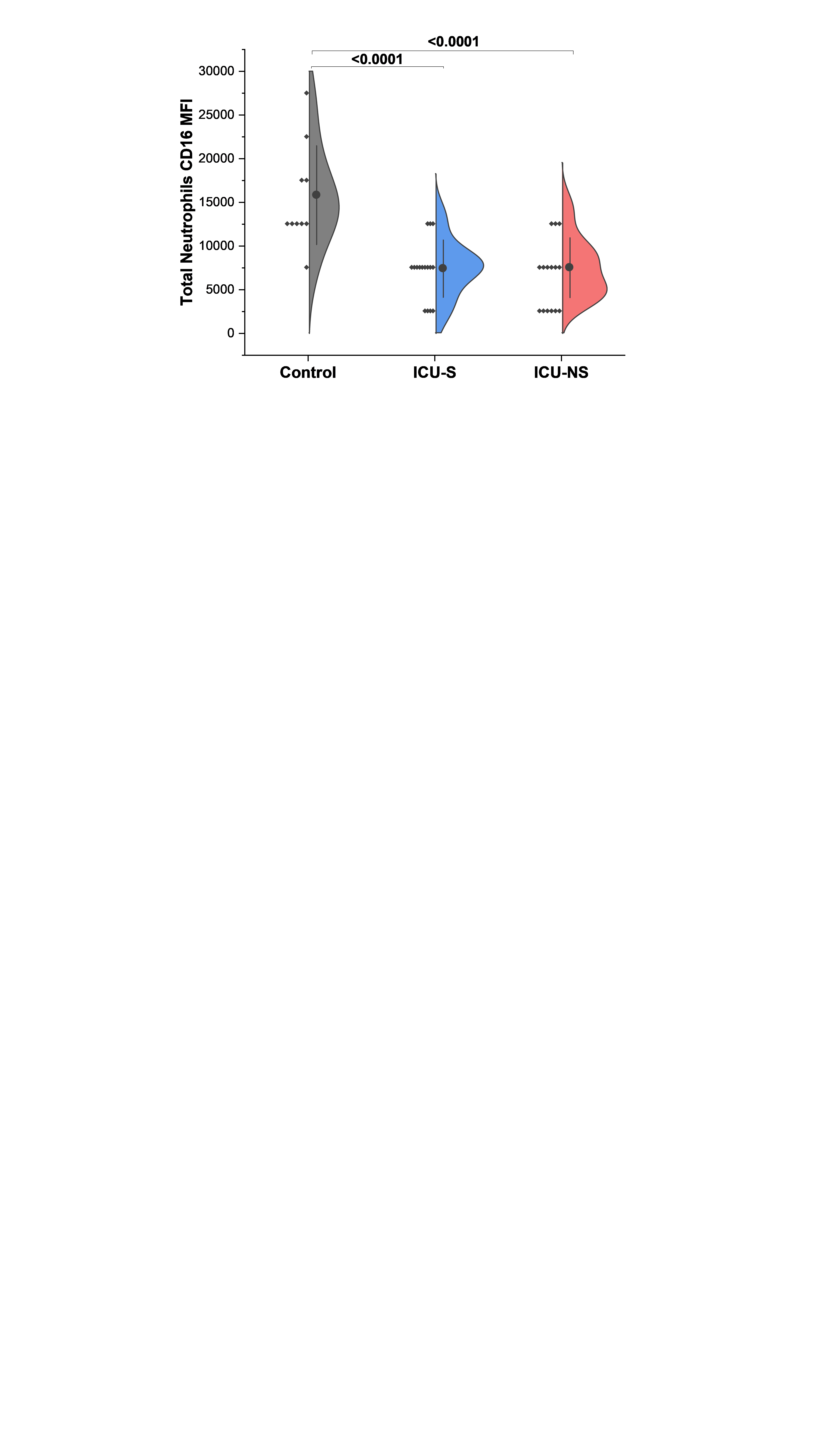

Supplement: Supplementary file 1 — Supplementary Figure 1 [file 41598_2026_38741_MOESM1_ESM.png]

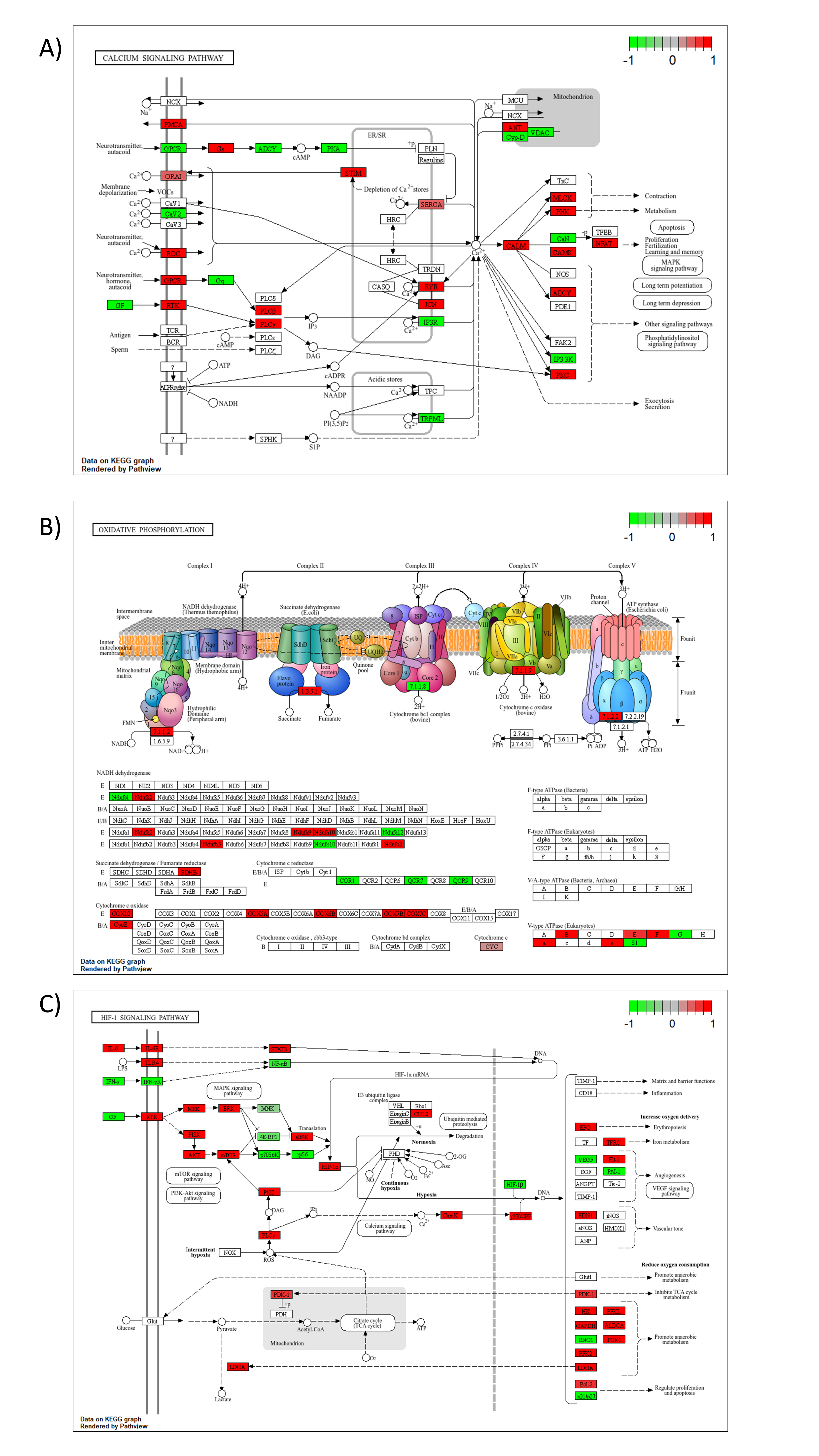

Supplement: Supplementary file 2 — Supplementary Figure 2 [file 41598_2026_38741_MOESM2_ESM.png]

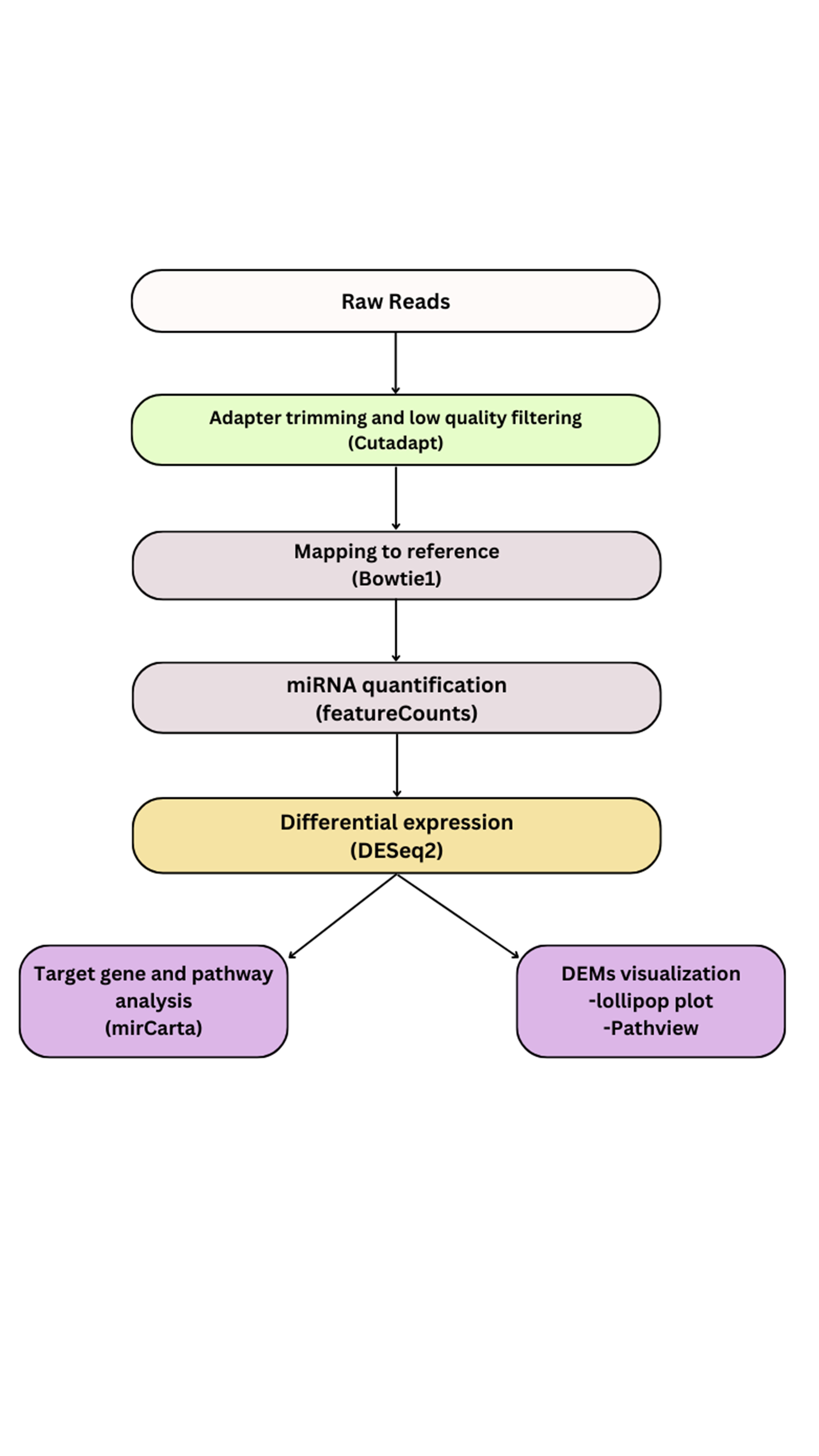

Supplement: Supplementary file 3 — Supplementary Figure 3 [file 41598_2026_38741_MOESM3_ESM.png]

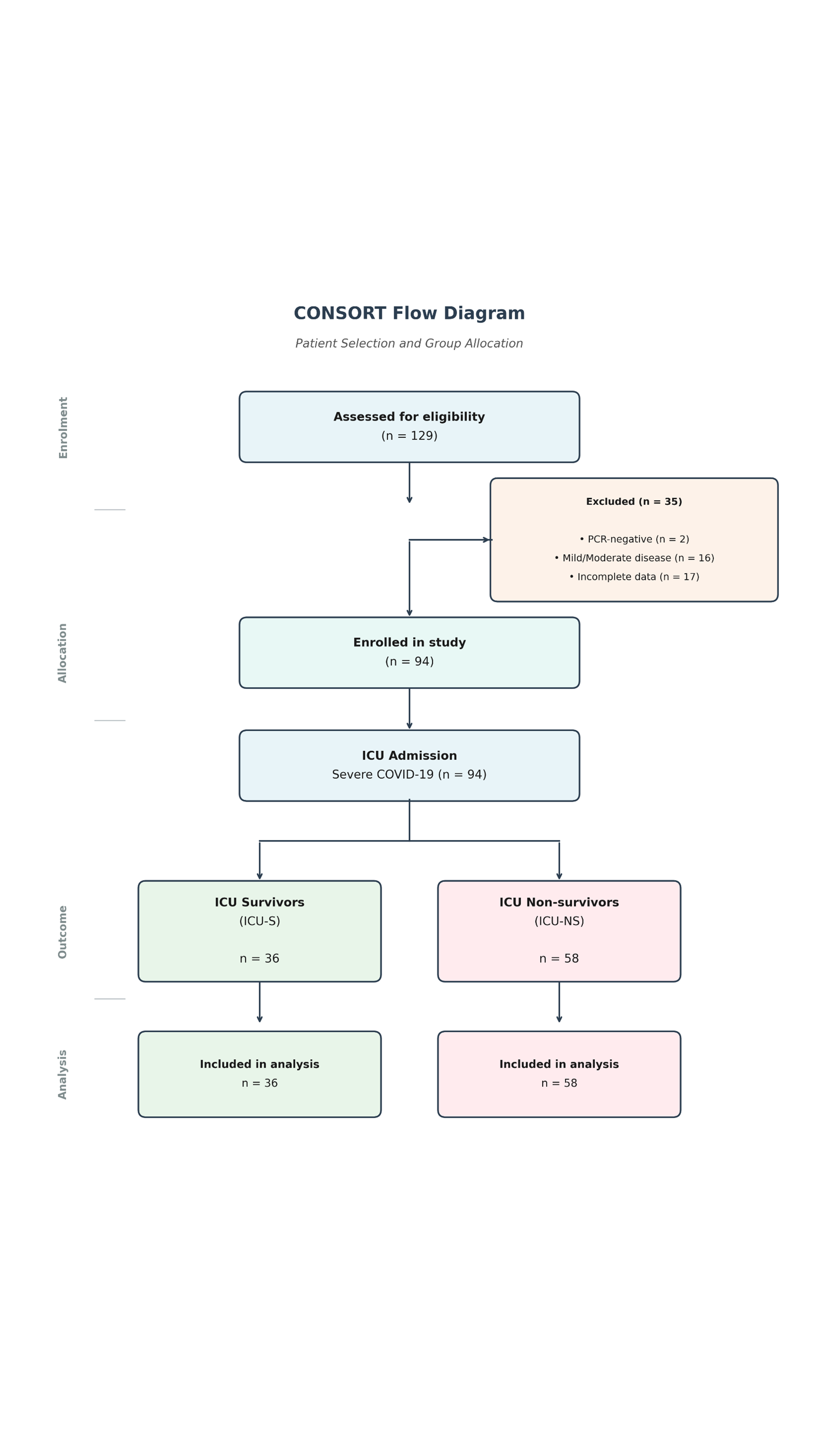

Supplement: Supplementary file 4 — Supplementary Figure 4 [file 41598_2026_38741_MOESM4_ESM.png]

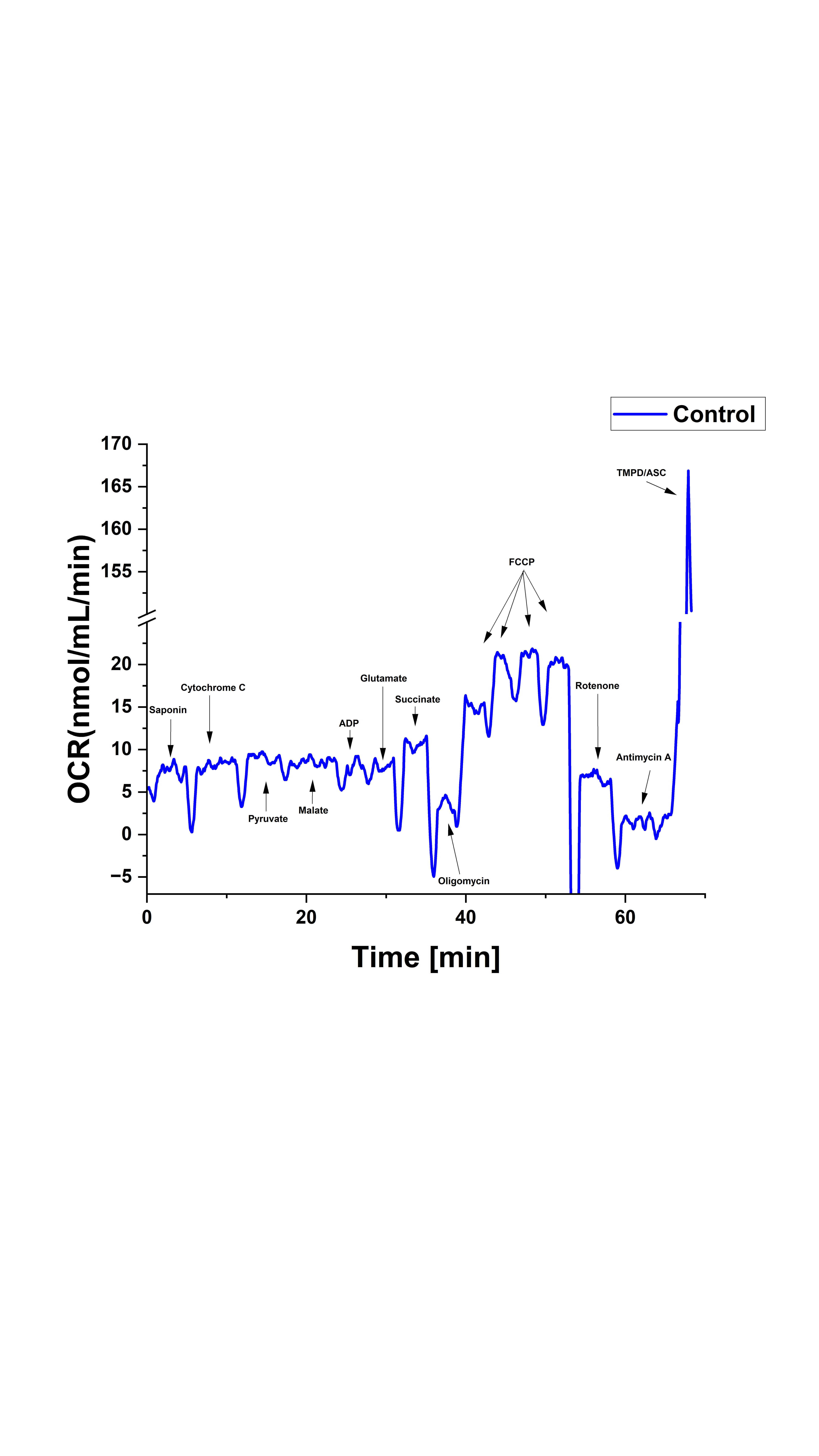

Supplement: Supplementary file 5 — Supplementary Figure 5 [file 41598_2026_38741_MOESM5_ESM.png]
