# Supplementary material for: Disruption of neutrophil homeostasis is associated with functional alterations in mitochondria of critically ill COVID−19 patients
Source: Sci Rep. 2026 Mar 1;16:7838. doi: 10.1038/s41598-026-38741-y (PMC12953904; doi:10.1038/s41598-026-38741-y)
Supplement: Supplementary file 6 — Supplementary Materials [file 41598_2026_38741_MOESM6_ESM.docx]

Supplementary Material

# Supplementary Figures

Supplementary Figure 1. Decreased CD16 MFI in neutrophils of ICU-COVID19 patients. A half violin plot showing decreased CD16 mean fluorescence intensity in neutrophils of ICU patients compared to controls (n= 10, 16 and, 15 for control, ICU-S and ICU-NS; respectively).

Supplementary Figure 2. KEGG pathway maps rendered with Pathview. (A) Calcium signalling pathway (hsa04020). (B) Oxidative phosphorylation (hsa00190). (C) Hypoxia-inducible factor 1 signalling pathway (hsa04066). Node colours indicate direction and magnitude of differential expression. Pathway maps were obtained from the Kyoto Encyclopedia of Genes and Genomes (Kanehisa et al., 2025; Kanehisa and Goto, 2000) and rendered using Pathview (Luo and Brouwer, 2013). Copyright © Kanehisa Laboratories. Reproduced with permission.

Supplementary Figure 3. Workflow steps for miRNA interaction analysis and visualization.

Supplementary Figure 4. CONSORT Diagram Flow Diagram showing patient selection and group allocation.

Supplementary Figure 5. Oxygraph showing changes in OCR following saponin and cytochrome c addition.

# Supplementary Tables

# Supplementary Table 1: Patients’ Demographics and Clinical Characteristics

|  | ICU-S n (%) | ICU-NS n (%) | p value |
| --- | --- | --- | --- |
| Male | 23 (63.9%) | 29 (50%) | 0.18 |
| Age | 61 (55-68) | 66 (52-73) | 0.14 |
| sO2 | 94 (83.5-97) | 90 (78-92) | **0.05** |
| Diabetes | 6 (16.7%) | 24 (41.4%) | **0.01** |
| Cardiovascular diseases | 11 (30.6%) | 22 (38%) | 0.46 |
| Cancer | 4 (11.1%) | 3 (5.2%) | 0.28 |
| Asthma | 2 (5.6%) | 3 (5.2%) | 0.93 |
| Insulin | 6 (16.7%) | 13 (22.4%) | 0.50 |
| Anticoagulant | 16 (44.4%) | 30 (51.7%) | 0.49 |
| Steroids | 11 (30.6%) | 29 (50%) | 0.06 |
| Hydroxychloroquine | 1 (2.87%) | 2 (3.4%) | 0.85 |
| IL6 inhibitors | 3 (8.3%) | 3 (5.2%) | 0.54 |
| Remdesivir | 3 (8.3%) | 15 (25.8%) | **0.03** |
| Ivermectin | 1 (2.8%) | 2 (3.4%) | 0.85 |
| Carbapenem antibiotics | 11 (30.6%) | 29 (50%) | 0.06 |
| Fluoroquinolone | 7 (19.4%) | 19 (32.75%) | 0.16 |
| Oxazolidinone antibiotic | 7 (19.4%) | 10 (17.2%) | 0.78 |

# Supplementary Table 2: Patients’ Laboratory Results

|  | ICU-S | ICU-NS | P-value |
| --- | --- | --- | --- |
| WBCs (×10^3^ /ml) | 9.5 (7.7-12.5) | 13.2 (8.5-17) | **0.02** |
| Platelets (10^6^ /ml) | 274.5±115 | 212.7±116 | **0.02** |
| Lymphocytes | 3.45 (1.05-8.92) | 2.6 (0.94-5.1) | 0.45 |
| Monocytes | 0.99 (0.42-5.02) | 1.26 (0.7-7.45) | 0.44 |
| INR | 1.12 (1.01-1.37) | 1.2 (1.1-1.37) | 0.25 |
| CRP (mg/L) | 56.5 (13.45-126.4) | 108.06 (51.7-202) | **0.008** |
| D**-**Dimer (mg/ml) | 0.98 (0.2-2.95) | 2.4 (1.18-5.76) | **0.004** |
| IL-6 (pg/ml) | 25 (4.24-885) | 31.8 (8.8-120.3) | 0.96 |
| Ferritin | 797 (476.5-954) | 1185 (583-2000) | **0.04** |
| Albumin | 2.75 (2.5-3.25) | 2.4 (2.1-3) | **0.02** |
| Hemoglobin (g/dl) | 11.7 (9.12-13.4) | 11.1 (9.5-12.2) | 0.52 |
| ALT (U/L) | 19 (13-48.5) | 22 (16-43) | 0.47 |
| AST (U/L) | 29 (18.5-34.5) | 40 (25-63) | 0.08 |
| Creatinine (mg/dl) | 0.96 (0.75-1.23) | 1.3 (0.8-2.65) | 0.11 |

**Supplementary Table 3****:** Genes interacting with the differentially expressed miRNAs between ICU-S and ICU-NS patients with their fold change.

| **Gene** | **Regulation status in Non-survived patients** | **Log2 (Fold Change)** |
| --- | --- | --- |
| **Apoptosis** |  |  |
| BCL-2 | ↑ | 0.764048 |
| BCL-XL | ↑ | 3.982416 |
| MCL-1 | ↑ | 0.035369 |
| CASP3 | ↓ | -1.701644 |
| Apaf-1 | ↓ | -1.436463 |
| p53 | ↓ | -0.89467 |
| Fas-L | ↓ | -5.068253 |
| TRAIL | ↓ | -1.436463 |
| FADD | ↓ | -1.528155 |
| HRK | ↓ | -1.5248445 |
| PUMA | ↑ | 3.9835126 |
| NOXA | ↑ | 1.6171734 |
| Bak | ↑ | 0.6392264 |
| **Calcium Signaling** |  |  |
| Cyp-D | ↓ | -2.001875 |
| VDAC | ↓ | -0.3221957 |
| ANT | ↑ | 2.9730513 |
| IP3R | ↓ | -2.96007 |
| SERCA | ↑ | 0.9539799 |
| STIM | ↑ | 2.9730513 |
| ORAI | ↑ | 0.437209 |
| **Complexes and their subunits** |  |  |
| **CXI** |  |  |
| Ndufs1 | ↓ | -1.436463 |
| Ndufs2 | ↑ | 1.143711 |
| Ndufa9 | ↑ | 4.0784555 |
| Ndufa10 | ↑ | 2.9730513 |
| Ndufa12 | ↓ | -1.5736087 |
| Ndufb5 | ↑ | 2.307626 |
| Ndufb10 | ↓ | -4.808774 |
| Ndufc2 | ↑ | 1.2768055 |
| **CXII** |  |  |
| SDHB | ↑ | 3.983513 |
| **CXIII** |  |  |
| QCR7 | ↓ | -1.669437561 |
| QCR9 | ↓ | -1.436463069 |
| **CXIV** |  |  |
| COX10 | ↑ | 3.7900525 |
| COX5A | ↑ | 4.0784555 |
| COX6B | ↑ | 2.3155216 |
| COX7B | ↑ | 2.973051265 |
| COX7C | ↑ | 3.98351261 |
| **HIF1A pathway** |  |  |
| HIF1A | ↑ | 2.973051265 |

Demographics and Laboratory Data per Experiment:

Flow Cytometry Neutrophils Counts

Supplementary Table 4. Patients’ Demographics and Clinical Characteristics.

|  | ICU-S n (%)  No. 32 | ICU-NS n (%)  No. 47 | p value |
| --- | --- | --- | --- |
| Male | 21 (65.6) | 26 (55.3) | 0.3 |
| Age | 60± 12 | 59.3± 15.7 | 0.8 |
| sO2 | 97 (59- 99) | 91 (59-97) | 0.16 |
| Diabetes | 6 (18.8) | 19 (40.4) | 0.05 |
| Cardiovascular diseases | 11 (34.4) | 17 (36.2) | 0.8 |
| Cancer | 4 (12.5) | 3 (6.4) | 0.4 |
| Asthma | 2 (6.3) | 3 (6.40 | 1 |
| Insulin | 5 (15.6) | 13 (27.7) | 0.2 |
| Anticoagulant | 16 (50) | 26 (55.3) | 0.6 |
| Steroids | 11 (34.4) | 27 (57.4) | **0.04** |
| Hydroxychloroquine | 1 93.1) | 2 (4.3) |  |
| IL6 inhibitors | 3 (9.4) | 2 (4.3) |  |
| Remdesivir | 2 (6.3) | 13 (27.7) | **0.02** |
| Ivermectin | 1 (3.1) | 1 (2.1) | 1 |
| Carbapenem antibiotics | 9 (28) | 26 (55.3) | **0.01** |
| Fluoroquinolone | 6 (18.8) | 16 (34) | 0.2 |
| Oxazolidinone antibiotic | 7 (21.9) | 10 (21.3) | 0.9 |

Both groups were comparable in age, gender distribution, and comorbidities. Diabetes was more prevalent among ICU-NS patients (40.4%) compared to ICU-S patients (18.8%) (p = 0.05). The use of steroids (p = 0.04), remdesivir (p = 0.02), and carbapenem antibiotics (p = 0.01) was significantly higher in ICU-NS patients. Other treatments, including IL-6 inhibitors, anticoagulants, and hydroxychloroquine, showed no statistically significant differences between the two groups (p > 0.05).

Flow Cytometry Neutrophils Counts

**Supplementary Table 5. Patients’ Laboratory Results.**

|  | ICU-S n (%)  No. 32 | ICU-NS n (%)  No.47 | P-value |
| --- | --- | --- | --- |
| WBCs (×10^3^ /ml) | 9.7 ±3.9 | 15.2± 6.9 | **0.007** |
| Platelets (10^6^ /ml) | 284± 124 | 206.8± 110 | **0.04** |
| Lymphocytes | 1± 0.8 | 2.2± (0.9-3.4) | 0.5 |
| Monocytes | - | - | - |
| INR | 1.2 (1-1.4) | 1 (0.07-1.09) | 0.2 |
| CRP (mg/L) | 23 (10.8- 34.5) | 156 (25.3-287) | **0.004** |
| D**-**Dimer (mg/ml) | 0.5 (0.3-3.6) | 1.4 (09-1.9) | **0.018** |
| IL-6 (pg/ml) | 3.7 (2.3- 4.7) | 23.8 (1.7-46) | 0.94 |
| Ferritin | 736± 235 | 732± 278 | **0.2** |
| Albumin | 2.9± 0.5 | 2.4± 0.4 | **0.001** |
| Hemoglobin (g/dl) | 11.7 ±0.4 | 11.8± 0.4 | - |
| ALT (U/L) | - | - | - |
| AST (U/L) | - | - | - |
| Creatinine (mg/dl) | 1 (0.3-6.8) | 1.4 (0.4-6.3) | 0.19 |

ICU-NS patients showed significantly higher white blood cell counts (p = 0.007), C-reactive protein (p = 0.004), D-dimer (p = 0.018), and lower platelet and albumin levels (p = 0.04 and p = 0.001, respectively). No significant differences were found in IL-6, ferritin, liver enzymes, or creatinine levels between both groups (p > 0.05).

Flow Cytometry CD16

Supplementary Table 6. Patients’ Demographics and Clinical Characteristics.

|  | ICU-S n (%)  No. 16 | ICU-NS n (%)  No.15 | p value |
| --- | --- | --- | --- |
| Male | 12 (75) | 5 (33.3) | 0.03 |
| Age | 53.6 ±11.1 | 36.6± 20 | 0.8 |
| sO2 | - | - | - |
| Diabetes | 2 (12.5) | 10 (66.7) | **0.003** |
| Cardiovascular diseases | 8 (50) | 9 (60) | 0.5 |
| Cancer | 3 (18.8) | 2 (13.3) | 1 |
| Asthma | 1 (6.3) | 2 (13.3) | 0.6 |
| Insulin | 2 (12.5) | 7 (46.7) | 0.054 |
| Anticoagulant | 10 (62.5) | 14 (93.3) | 0.08 |
| Steroids | 5 (31.3) | 12 (80) | **0.01** |
| Hydroxychloroquine | 0 | 0 | - |
| IL6 inhibitors | 0 | 1 (6.7) | 0.4 |
| Remdesivir | 3 (18.8) | 5 (33.3) | 0.4 |
| Ivermectin | 1 (6.3) | 1 (6.7) | 1 |
| Carbapenem antibiotics | 7 (43.8) | 11 (73.3) | 0.14 |
| Fluoroquinolone | 5 (31.3) | 8 (53.3) | 0.2 |
| Oxazolidinone antibiotic | 3 (18.8) | 4 (26.7) | 0.68 |

There was a significantly higher proportion of males in the ICU-survivor group compared to the ICU-non-survivor group (75% vs. 33.3%, *p* = 0.03). Diabetes was significantly more frequent among non-survivors (66.7%) than survivors (12.5%, *p* = 0.003). Steroid use was also significantly higher in the non-survivor group (80%) compared to survivors (31.3%, *p* = 0.01).

Flow Cytometry CD16

**Supplementary Table 7. Patients’ Laboratory Results.**

|  | ICU-S n (%)  No. 16 | ICU-NS n (%)  No.15 | P-value |
| --- | --- | --- | --- |
| WBCs (×10^3^ /ml) | 8.9± 3.2 | 17.4± 10.5 | 0.05 |
| Platelets (10^6^ /ml) | 394± 144.9 | 123.9± 111.6 | 0.19 |
| Lymphocytes | - | - | - |
| Monocytes | - | - | - |
| INR | 1.1± 0.16 | 1.1± 0.16 | 0.3 |
| CRP (mg/L) | 23.4± 35.2 | 155± 81 | 0.25 |
| D**-**Dimer (mg/ml) | 1 (0.9-3.17) | 2 (1.3-19.1) | 0.17 |
| IL-6 (pg/ml) | - | - | - |
| Ferritin | - | - | **-** |
| Albumin | - | - | **-** |
| Hemoglobin (g/dl) | 9.9± 2.7 | 10± 1.8 | 0.9 |
| ALT (U/L) | - | - | - |
| AST (U/L) | - | - | **-** |
| Creatinine (mg/dl) | 0.4± 0.3 | 1.7± 1.5 | 0.6 |

No statistically significant differences were observed between ICU survivors and non-survivors. Although non-survivors tended to have higher WBC, CRP, D-dimer, and creatinine levels and lower platelet counts, these differences did not reach statistical significance (*p* > 0.05).

**Fluorescent Imaging Annexin**

Supplementary Table 8. Patients’ Demographics and Clinical Characteristics.

|  | ICU-S n (%)  No. 6 | ICU-NS n (%)  No.10 | p value |
| --- | --- | --- | --- |
| Male | 5 (83.3) | 5 (50) | 0.3 |
| Age | 53± 11 | 57± 12.8 | 0.2 |
| sO2 | - | - | - |
| Diabetes | 1 (16.7) | 6 (60) | 0.14 |
| Cardiovascular diseases | 0 | 4 (40) | 0.2 |
| Cancer | 1 (16.7) | 0 | 0.3 |
| Asthma | 1 (16.7) | 1 (10) | 1 |
| Insulin | 2 (33.3) | 1 (10) | 0.5 |
| Anticoagulant | 2 (33.3) | 4 (40) | 1 |
| Steroids | 2 (33.3) | 6 (60) | 0.6 |
| Hydroxychloroquine | 0 | 0 | 0.2 |
| IL6 inhibitors | 1 (16.7) | 1 (10) | 1 |
| Remdesivir | 0 | 4 (40) | - |
| Ivermectin | 0 | 1 (10) | 1 |
| Carbapenem antibiotics | 3 (50) | 6 (60) | 1 |
| Fluoroquinolone | 0 | 2 (20) | 0.5 |
| Oxazolidinone antibiotic | 1 (16.7) | 0 | 0.3 |

Both groups were comparable in terms of age, gender, and comorbidities. Diabetes and cardiovascular diseases were more frequent among ICU-NS patients, although the differences were not statistically significant (p = 0.14 and p = 0.2, respectively). The use of steroids, insulin, anticoagulants, and broad-spectrum antibiotics such as carbapenems was higher among ICU-NS patients, but no significant associations were found (p > 0.05).

**Fluorescent Imaging Annexin**

Supplementary Table 9. Patients’ Laboratory Results.

|  | ICU-S n (%)  No. 6 | ICU-NS n (%)  No.10 | P-value |
| --- | --- | --- | --- |
| WBCs (×10^3^ /ml) | 10.9± 3.3 | 12.5± 4.3 | 0.2 |
| Platelets (10^6^ /ml) | 142± 25 | 169± 75 | 0.06 |
| Lymphocytes | - | - | - |
| Monocytes | - | - | - |
| INR | 1± 0.01 | 1.2± 0.18 | 0.1 |
| CRP (mg/L) | 75.8± 58 | 121.4± 79 | 0.3 |
| D**-**Dimer (mg/ml) | 1.5 (0.2-5.2) | 4.3 (1.1-11) | 0.2 |
| IL-6 (pg/ml) | - | - | - |
| Ferritin | 219.5 (218-221) | 997.5 (139-2000) | 0.16 |
| Albumin | 3± 0.6 | 2.5± 0.4 | 0.5 |
| Hemoglobin (g/dl) | 11.3± 0.5 | 11.5± 2.4 | 0.4 |
| ALT (U/L) | - | - | - |
| AST (U/L) | - | - | - |
| Creatinine (mg/dl) | 1 (0.7-2.2) | 1.5 (0.3-5.6) | 0.8 |

Although ICU-NS patients showed higher levels of inflammatory and coagulation markers, including CRP, D-dimer, and ferritin, none of these differences reached statistical significance (p > 0.05). Similarly, no significant variations were observed in hematological or biochemical parameters such as WBCs, platelets, hemoglobin, albumin, ALT, AST, or creatinine between both groups.

**Fluorescent Imaging Cleaved Caspase 3**

Supplementary Table 10. Patients’ Demographics and Clinical Characteristics.

|  | ICU-S n (%)  No. 2 | ICU-NS n (%)  No.5 | p value |
| --- | --- | --- | --- |
| Male | 0 | 1 (20) | 1 |
| Age | 61±5.6 | 66.3± 10.7 | 0.4 |
| sO2 | 97.5± 0.7 | 82.67± 1.1 | **0.006** |
| Diabetes | 1 (50) | 3 (60) | 1 |
| Cardiovascular diseases | 1 (50) | 3 (60) | 1 |
| Cancer | 1 (50) | 1 (20) | 1 |
| Asthma | 0 | 0 | - |
| Insulin | 1 (50) | 2 (40) | 1 |
| Anticoagulant | 2 (100) | 5 (100) | - |
| Steroids | 1 (50) | 5 (100) | 0.28 |
| Hydroxychloroquine | 0 | 0 | - |
| IL6 inhibitors | 0 | 0 | - |
| Remdesivir | 0 | 4 (80) | 0.14 |
| Ivermectin | 0 | 0 | - |
| Carbapenem antibiotics | 1 (50) | 4 (80) | 1 |
| Fluoroquinolone | 0 | 4 (80) | 0.14 |
| Oxazolidinone antibiotic | 1 (50) | 3 (60) | 1 |

Most variables showed no statistically significant differences between ICU survivors and non-survivors (*p* > 0.05). The only significant finding was a lower mean oxygen saturation (sO₂) in non-survivors (82.7 ± 1.1) compared to survivors (97.5 ± 0.7) (*p* = 0.006). Other factors, including age, comorbidities, and use of medications, were comparable between the two groups.

Fluorescent Imaging Cleaved Caspase 3

Supplementary Table 11. Patients’ Laboratory Results.

|  | ICU-S n (%)  No. 2 | ICU-NS n (%)  No.5 | P-value |
| --- | --- | --- | --- |
| WBCs (×10^3^ /ml) | 8.9± 0.84 | 16.7± 1.1 | 0.27 |
| Platelets (10^6^ /ml) | 231.5± 101 | 206.6± 43.8 | 0.6 |
| Lymphocytes | - | - | - |
| Monocytes | - | - | - |
| INR | 1.1 | 1.2 (1-2.6) | 0.3 |
| CRP (mg/L) | 61± 5.6 | 178.3± 95.9 | 0.3 |
| D**-**Dimer (mg/ml) | - | - | - |
| IL-6 (pg/ml) | - | - | - |
| Ferritin | - | - | - |
| Albumin | 2.5± 0.07 | 2.5± 0.36 | 0.6 |
| Hemoglobin (g/dl) | 10.9± 0.2 | 12.6± 0.9 | 0.7 |
| ALT (U/L) | - | - | - |
| AST (U/L) | - | - | **-** |
| Creatinine (mg/dl) | 0.65 (0.3-1) | 0.8 (0.78-3.9) | 0.4 |

No statistically significant differences were observed between ICU survivors and non-survivors across all measured parameters (*p* > 0.05). Although non-survivors had higher mean WBC and CRP levels, these differences did not reach significance. Albumin, hemoglobin, and creatinine levels were also comparable between the two groups.

Flow Cytometry Fluo4 and TMRM and Annexin

Supplementary Table 12. Patients’ Demographics and Clinical Characteristics.

|  | ICU-S n (%)  No. 16 | ICU-NS n (%)  No.28 | p value |
| --- | --- | --- | --- |
| Male | 11 (68.8) | 19 (37.9) | 0.9 |
| Age | 72 (67-77) | 66.5 (60-73) | 0.78 |
| sO2 | 76 (66-89) | 89 (86-92) | 0.32 |
| Diabetes | 3 (18.8) | 7 (25) | 0.7 |
| Cardiovascular diseases | 2 (12.5) | 7 (25) | 0.45 |
| Cancer | 0 | 0 | - |
| Asthma | 1 (6.3) | 1 (3.6) | 1 |
| Insulin | 3 (18.8) | 4 (14.3) | 0.69 |
| Anticoagulant | 4 (25) | 9 (32) | 0.73 |
| Steroids | 5 (31.3) | 10 (35.7) | 0.76 |
| Hydroxychloroquine | 1 (6.3) | 2 (7.1) | 1 |
| IL6 inhibitors | 3 (18.8) | 1 (3.6) | 0.12 |
| Remdesivir | 0 | 4 (14.3) | 0.28 |
| Ivermectin | 0 | 0 | - |
| Carbapenem antibiotics | 3 (18.8) | 12 (42.9) | 0.18 |
| Fluoroquinolone | 2 (12.5) | 5 (17.9) | 1 |
| Oxazolidinone antibiotic | 3 (188) | 3 (10.7) | 0.65 |

Table 12 summarizes the demographic and clinical characteristics of the patients studied. A total of 44 patients were included, divided into ICU-S (n = 16) and ICU-NS (n = 28) groups. There were no statistically significant differences between both groups in terms of demographic or clinical parameters.

Flow Cytometry Fluo4 and TMRM

Supplementary Table 13. Patients’ Laboratory Results.

|  | ICU-S n (%)  No. 16 | ICU-NS n (%)  No.28 | P-value |
| --- | --- | --- | --- |
| WBCs (×10^3^ /ml) | 11.3 ±4.6 | 13.7 ±0.43 | 0.34 |
| Platelets (10^6^ /ml) | 239.5 ±36 | 224 ±99 | 0.33 |
| Lymphocytes | 0.8 (0.6-1.1) | 0.6 (0.25-0.97) | 0.64 |
| Monocytes | 0.7 (0.34-1.1) | 0.34 (0.2-0.48) | 0.15 |
| INR | 1.3 (1.2-1.4) | 1.1 (1.07-1.6) | 0.37 |
| CRP (mg/L) | 34.5 (13.6-144) | 25.3 (12-287) | **0.02** |
| D**-**Dimer (mg/ml) | 1.1 (0.49-5.2) | 0.9 (0.44-1.9) | **0.03** |
| IL-6 (pg/ml) | 25 (3.7-83.8) | 7 (1.7-46) | 0.92 |
| Ferritin | 797 (477-938) | 535 (313-929) | **0.03** |
| Albumin | 2.9±0.3 | 2.4 ±0.4 | **0.01** |
| Hemoglobin (g/dl) | 13.8 (11.7-14.1) | 11.9 (11.5-12.1) | 0.06 |
| ALT (U/L) | 9 (8-20) | 20 (20-43) | 0.6 |
| AST (U/L) | 17 (17-40) | 34 (31-56) | **0.03** |
| Creatinine (mg/dl) | 0.9 (0.6-1.1) | 1.0 (0.5-1.2) | 0.1 |

Table 13 presents the laboratory findings of the studied patients. No statistically significant differences were found between the ICU-S and ICU-NS groups in most hematological and biochemical parameters, except for a few markers that showed notable variations. Among inflammatory and biochemical markers, **CRP, D-dimer, ferritin, albumin,** and **AST** levels showed statistically significant differences. CRP levels were significantly higher in ICU-S patients [34.5 (13.6–144) mg/L] than ICU-NS [25.3 (12–287) mg/L] (p = 0.02). Similarly, D-dimer [1.1 (0.49–5.2) mg/ml vs. 0.9 (0.44–1.9) mg/ml, p = 0.03] and ferritin [797 (477–938) ng/ml vs. 535 (313–929) ng/ml, p = 0.03] were significantly elevated among ICU-S patients. In contrast, serum albumin levels were significantly lower in the ICU-S group (2.9 ± 0.3 g/dl) compared to ICU-NS (2.4 ± 0.4 g/dl, p = 0.01).

Fluorescent Imaging MICU1/ Cyclophilin D

Supplementary Table 14. Patients’ Demographics and Clinical Characteristics.

|  | ICU-S n (%)  No. 2 | ICU-NS n (%)  No.7 | p value |
| --- | --- | --- | --- |
| Male | 0 | 2 (28.6) | 1 |
| Age | 61±5.6 | 64± 14.2 | 0.7 |
| sO2 | 97.5± 0.7 | 92.4± 1.1 | **0.01** |
| Diabetes | 1 (50) | 4 (57) | 1 |
| Cardiovascular diseases | 1 (50) | 3 (43) | 1 |
| Cancer | 1 (50) | 1 (14.3) | 0.4 |
| Asthma | 0 | 0 | NA |
| Insulin | 1 (50) | 3 (43) | 1 |
| Anticoagulant | 2 (100) | 7 (100) | NA |
| Steroids | 1 (50) | 7 (100) | 0.2 |
| Hydroxychloroquine | 0 | 0 | NA |
| IL6 inhibitors | 0 | 0 | NA |
| Remdesivir | 0 | 6 (85.7) | 0.08 |
| Ivermectin | 0 | 0 | NA |
| Carbapenem antibiotics | 1 (50) | 6 (85.7) | 0.4 |
| Fluoroquinolone | 0 | 5 (71.4) | 0.16 |
| Oxazolidinone antibiotic | 1 (50) | 4 (57) | 1 |

Most parameters showed no statistically significant differences between ICU survivors and non-survivors (*p* > 0.05). The only significant finding was a lower mean oxygen saturation (sO₂) in non-survivors (92.4 ± 1.1) compared to survivors (97.5 ± 0.7) (*p* = 0.01). Other characteristics, including age, comorbidities, and medication use, were comparable between both groups.

Fluorescent Imaging MICU1/ Cyclophilin D

Supplementary Table 15. Patients’ Laboratory Results.

|  | ICU-S n (%)  No. 2 | ICU-NS n (%)  No.7 | P-value |
| --- | --- | --- | --- |
| WBCs (×10^3^ /ml) | 8.9 (8.3-9.5) | 18.3 (16-23) | 0.14 |
| Platelets (10^6^ /ml) | 231.5± 101 | 279.6± 116 | 0.8 |
| Lymphocytes | - | - | - |
| Monocytes | - | - | - |
| INR | 1 | 1.4± 0.6 | 0.37 |
| CRP (mg/L) | 121± 77.7 | 159± 86 | 0.6 |
| D**-**Dimer (mg/ml) | - | - | - |
| IL-6 (pg/ml) | - | - | - |
| Ferritin | - | - | - |
| Albumin | 2.5± 0.07 | 2.3± 0.4 | 0.4 |
| Hemoglobin (g/dl) | 11± 0.2 | 9.2± 1.9 | 0.9 |
| ALT (U/L) | - | - | - |
| AST (U/L) | - | - | **-** |
| Creatinine (mg/dl) | 0.65 ± 0.5 | 92.4± 1.1 | 0.29 |

No statistically significant differences were observed between ICU survivors and non-survivors across all tested parameters (*p* > 0.05). Although non-survivors exhibited higher mean WBC counts and CRP levels, these differences did not reach statistical significance. Other laboratory markers, including platelets, INR, albumin, hemoglobin, liver enzymes, and creatinine, were comparable between both groups.

Seahorse analyzer

Supplementary Table 16. Patients’ Demographics and Clinical Characteristics.

|  | ICU-S n (%)  No. 15 | ICU-NS n (%)  No.16 | p value |
| --- | --- | --- | --- |
| Male | 12 (70.6) | 7 (50) | 0.2 |
| Age | 64± 9 | 67.3± 8 | 0.3 |
| sO2 | 91± 10 | 87.5± 6.9 | 0.4 |
| Diabetes | 4 (23.5) | 7 (50) | 0.1 |
| Cardiovascular diseases | 6 (35.3) | 5 (35.7) | 0.9 |
| Cancer | 2 (11.8) | 0 | 0.4 |
| Asthma | 2 (11.8) | 2 (14.3) | 1 |
| Insulin | 3 (17.6) | 3 (21.4) | 1 |
| Anticoagulant | 6 (35.3) | 9 (64.3) | 0.1 |
| Steroids | 6 (35.3) | 8 (57.1) | 0.2 |
| Hydroxychloroquine | 1 (5.9) | 1 (7.1) | 1 |
| IL6 inhibitors | 2 (11.8) | 1 (7.1) | 1 |
| Remdesivir | 0 | 5 (35.7) | **0.01** |
| Ivermectin | 1 (5.9) | 1 (7.1) | 1 |
| Carbapenem antibiotics | 5 (29.4) | 7 (50) | 0.2 |
| Fluoroquinolone | 5 (29.4) | 3 (21.4) | 0.6 |
| Oxazolidinone antibiotic | 3 (17.6) | 4 (26.6) | 0.6 |

Most parameters showed no statistically significant differences between ICU survivors and non-survivors (*p* > 0.05). The only significant finding was a higher frequency of remdesivir use among non-survivors (35.7%) compared to survivors (0%) (*p* = 0.01). Other variables, including age, gender, comorbidities, oxygen saturation, and use of other medications, were comparable between both groups.

Seahorse analyzer

Supplementary Table 17. Patients’ Laboratory Results.

|  | ICU-S n (%)  No. 15 | ICU-NS n (%)  No.16 | P-value |
| --- | --- | --- | --- |
| WBCs (×10^3^ /ml) | 8.8± 2.4 | 15.9± 9.2 | **0.01** |
| Platelets (10^6^ /ml) | 255± 127 | 236.7± 107 | 0.7 |
| Lymphocytes | 1.9 (0.6-24.5) | 0.9 (0.2-15) | 0.7 |
| Monocytes | 0.5 (0.11-2.4) | 0.9 (0.2-11) | 0.6 |
| INR | 1.1± 0.19 | 1.1± 0.15 | 0.8 |
| CRP (mg/L) | 33.6 (1.9-161.8) | 64.4 (12-305) | 0.06 |
| D**-**Dimer (mg/ml) | 0.4 (0.17-3.7) | 1 (0.19-19.1) | 0.7 |
| IL-6 (pg/ml) | 4.2 (2.3-1830) | 31.8 (838-235) | 0.6 |
| Ferritin | 660.2± 407 | 1132.7± 939.6 | 0.2 |
| Albumin | 2.9± 0.7 | 2.4± 0.5 | 0.2 |
| Hemoglobin (g/dl) | 12± 1.5 | 11.9± 1.5 | 0.9 |
| ALT (U/L) | 38.6± 31 | 40± 25.6 | 0.9 |
| AST (U/L) | 27.5± 7.4 | 53.6± 26.5 | 0.08 |
| Creatinine (mg/dl) | 1.1 (0.5-5.9) | 1.4 (0.48-6.3) | 0.6 |

A significantly higher WBC count was observed among ICU non-survivors compared to survivors (15.9 ± 9.2 vs. 8.8 ± 2.4 ×10³/ml, *p* = 0.01). Other laboratory parameters, including platelets, lymphocytes, CRP, D-dimer, IL-6, ferritin, liver enzymes, and creatinine, showed no statistically significant differences between the two groups (*p* > 0.05), although CRP and AST levels tended to be higher among non-survivors.

Oroborous (Respirometry)

Supplementary Table 18. Patients’ Demographics and Clinical Characteristics.

|  | ICU-S n (%)  No. 9 | ICU-NS n (%)  No.28 | P-value |
| --- | --- | --- | --- |
| Male | 6 (66.7) | 16 (57.1) | 0.7 |
| Age | 73.5±5 | 60.5± 4.9 | 0.18 |
| sO2 | 86± 18.5 | 78± 10.5 | 0.3 |
| Diabetes | 1 (11.1) | 8 (28.6) | 0.4 |
| Cardiovascular diseases | 0 | 8 | 0.15 |
| Cancer | 0 | 0 | - |
| Asthma | 1 (11.1) | 1 (3.6) | 0.4 |
| Insulin | 1 (11.1) | 2 (7.1) | 1 |
| Anticoagulant | 1 (11.1) | 9 (32.1) | 0.39 |
| Steroids | 2 (22.2) | 10 (35.7) | 0.6 |
| Hydroxychloroquine | 0 | 2 (7.1) | 1 |
| IL6 inhibitors | 1 (11.1) | 2 (7.1) | 1 |
| Remdesivir | 0 | 6 (21.4) | 0.3 |
| Ivermectin | 0 | 1 | 1 |
| Carbapenem antibiotics | 2 (22.2) | 11 (39.3) | 0.44 |
| Fluoroquinolone | 0 | 4 (14.3) | 0.5 |
| Oxazolidinone antibiotic | 0 | 2 (7.1) | 1 |

No statistically significant differences were observed between ICU survivors and non-survivors in terms of age, gender, oxygen saturation, comorbidities, or treatment regimens. Both groups showed comparable distributions of diabetes, cardiovascular diseases, and medication use (*p* > 0.05).

**Oroborous (Respirometry)**

**Supplementary Table 19. Patients’ Laboratory Results.**

|  | ICU-S n (%)  No. 9 | ICU-NS n (%)  No.28 | P-value |
| --- | --- | --- | --- |
| WBCs (×10^3^ /ml) | 11± 4.6 | 13.5± 7.1 | 0.3 |
| Platelets (10^6^ /ml) | 182 (160-321) | 208 (80-356) | 0.4 |
| Lymphocytes | 0.8 (0.6-1.1) | 0.8 (0.2-2.4) | 0.19 |
| Monocytes | 0.7 (0.3-1.1) | 0. (0.2-12.4) | 0.8 |
| INR | 1.3 (1.2-1.4) | 1.1 (1-1.2) | 0.27 |
| CRP (mg/L) | 89.2± 77.4 | 104.2± 124 | 0.2 |
| D**-**Dimer (mg/ml) | 2.8± 3.3 | 1.7± 2 | 0.3 |
| IL-6 (pg/ml) | 14.3 (3.74-25) | 54.7 (7-120.3) | 0.6 |
| Ferritin | 637 (477-797) | 652.8 (313-1315) | **0.03** |
| Albumin | 3± 0.5 | 2.3± 0.3 | 0.6 |
| Hemoglobin (g/dl) | 12.9 (11.7-14.1) | 11.4 (10.9-12.1) | 0.14 |
| ALT (U/L) | 8.5 (8-9) | 19.5 (11-43) | 0.26 |
| AST (U/L) | ± | 38± 17.2 | 0.25 |
| Creatinine (mg/dl) | 1.2± 0.6 | 2.5± 1.8 | 0.5 |

Most parameters, including WBCs, platelets, lymphocytes, CRP, D-dimer, IL-6, albumin, hemoglobin, and liver and renal function markers, showed no statistically significant differences between the two groups (*p* > 0.05). The only significant difference was observed in serum ferritin levels, which were higher in non-survivors compared to survivors (*p* = 0.03), indicating a possible association between elevated ferritin and poorer outcomes.

Electron Microscopy

Supplementary Table 20. Patients’ Demographics and Clinical Characteristics.

|  | ICU-S n (%)  No. 5 | ICU-NS n (%)  No.4 | p value |
| --- | --- | --- | --- |
| Male | 2 (40) | 2 (50) | 1 |
| Age | 67± 8.5 | 69± 7 | 0.27 |
| Diabetes | 0 | 1 (25) | 0.44 |
| Cardiovascular diseases | 0 | 1 (25) | 0.44 |
| Cancer | 0 | 0 | - |
| Asthma | 0 | 0 | - |
| Insulin | 0 | 0 | - |
| Anticoagulant | 2 (40) | 2 (50) | 1 |
| Steroids | 0 | 3 (75) | 0.48 |
| Hydroxychloroquine | 0 | 0 | - |
| IL6 inhibitors | 1 | 0 | 1 |
| Remdesivir | 0 | 2 (50) | 0.16 |
| Ivermectin | 0 | 0 | - |
| Carbapenem antibiotics | 1 (20) | 2 (50) | 0.5 |
| Fluoroquinolone | 0 | 0 | - |
| Oxazolidinone antibiotic | 0 | 0 | - |

No statistically significant differences were observed between ICU survivors and non-survivors (*p* > 0.05). Both groups were comparable in terms of age, gender, comorbidities, and medication use. None of the assessed variables, including diabetes, cardiovascular diseases, or treatment regimens, showed a significant association with survival outcomes.

Electron Microscopy

**Supplementary Table 21. Patients’ Laboratory Results.**

|  | ICU-S n (%)  No. 5 | ICU-NS n (%)  No.4 | P-value |
| --- | --- | --- | --- |
| WBCs (×10^3^ /ml) | 10 (8.3-11.8) | 10 (9.7-0.57) | 0.14 |
| Platelets (10^6^ /ml) | 323± 3.5 | 257± 14.8 | 0.4 |
| Lymphocytes | - | - | - |
| Monocytes | - | - | - |
| INR | 1±0.04 | 1.3± 0.3 | 0.26 |
| CRP (mg/L) | 119± 45 | 206± 16.8 | **0.04** |
| D**-**Dimer (mg/ml) | 0.59 (0.18-1.5) | 4.9 (1.1-8.8) | 0.18 |
| IL-6 (pg/ml) | - | - | - |
| Ferritin | 1141± 812 | 833± 497 | 0.58 |
| Albumin | 3± 1.06 | 2.8± 0.56 | 0.9 |
| Hemoglobin (g/dl) | 13.1±1.9 | 12.1± 1.6 | 0.75 |
| ALT (U/L) | - | - | - |
| AST (U/L) | - | - | **-** |
| Creatinine (mg/dl) | 1.6± 0.7 | 1.6± 0.03 | 0.9 |

Most laboratory parameters did not differ significantly between ICU survivors and non-survivors (*p* > 0.05). However, CRP levels were significantly higher in non-survivors compared to survivors (206 ± 16.8 vs. 119 ± 45 mg/L, *p* = 0.04), indicating greater inflammatory activity among non-survivors. Other markers, including WBCs, platelets, INR, D-dimer, ferritin, and liver and kidney function tests, showed no significant differences.

Fluorescent Imaging Mitotracker and Fluo4

Supplementary Table 22. Patients’ Demographics and Clinical Characteristics.

|  | ICU-S n (%)  No. 5 | ICU-NS n (%)  No.9 | p value |
| --- | --- | --- | --- |
| Male | 4 (80) | 4 (44.4) | 0.3 |
| Age | 59.6± 11 | 60.8± 9.8 | 0.15 |
| sO2 | 98± 1.4 | 71± 10.8 | **0.04** |
| Diabetes | - | - | - |
| Cardiovascular diseases | 0 | 3 (33.3) | - |
| Cancer | 0 | 0 | - |
| Asthma | 1 (20) | 1 (11.1) | 1 |
| Insulin | 1 (20) | 0 | 0.3 |
| Anticoagulant | 1 (20) | 3 (33.3) | 1 |
| Steroids | 1 (20) | 5 (55.6) | 0.3 |
| Hydroxychloroquine | 0 | 0 | - |
| IL6 inhibitors | 1 (20) | 1 (11.1) | 1 |
| Remdesivir | 0 | 4 (44.4) | 0.2 |
| Ivermectin | 0 | 1 (11.1) | 1 |
| Carbapenem antibiotics | 2 (40) | 5 (55.6) | - |
| Fluoroquinolone | 0 | 2 (22.2) | 0.5 |
| Oxazolidinone antibiotic | - | - | - |

The two groups were comparable regarding age, gender distribution, and comorbidities. Mean oxygen saturation (sO₂) was significantly lower among ICU-NS patients (71 ± 10.8%) compared with ICU-S patients (98 ± 1.4%, p = 0.04). No significant differences were found in the use of insulin, steroids, anticoagulants, or other therapeutic agents, including IL-6 inhibitors, remdesivir, or carbapenem antibiotics (p > 0.05).

Fluorescent Imaging Mitotracker

**Supplementary Table 23. Patients’ Laboratory Results.**

|  | ICU-S n (%)  No. 5 | ICU-NS n (%)  No.9 | P-value |
| --- | --- | --- | --- |
| WBCs (×10^3^ /ml) | 9.5± 3 | 14.3± 5 | 0.26 |
| Platelets (10^6^ /ml) | 218± 77 | 158± 85 | 0.2 |
| Lymphocytes | - | - | - |
| Monocytes | - | - | - |
| INR | - | - | - |
| CRP (mg/L) | 104.8± 54 | 119.3± 89 | 0.5 |
| D**-**Dimer (mg/ml) | 1.5 (0.24-3.7) | 1.7 (1.1-8.8) | 0.5 |
| IL-6 (pg/ml) | - | - | - |
| Ferritin | 221 (218-439) | 1185 (139-20000) | 0.12 |
| Albumin | 2.9± 0.9 | 2.2± 0.4 | 0.6 |
| Hemoglobin (g/dl) | 12.2± 1.5 | 11.5± 2.7 | 0.8 |
| ALT (U/L) | - | - | - |
| AST (U/L) | - | - | **-** |
| Creatinine (mg/dl) | 1.6± 0.8 | 2.7± 2.5 | 0.9 |

No statistically significant differences were detected between the two groups in white blood cell count, platelet count, C-reactive protein (CRP), D-dimer, ferritin, or other biochemical parameters (p > 0.05). Although ICU-NS patients tended to have higher ferritin and creatinine levels and lower albumin and hemoglobin levels, these differences did not reach statistical significance.

Fluorescent Imaging Mitosox

Table 24. Patients’ Demographics and Clinical Characteristics.

|  | ICU-S n (%)  No. 5 | ICU-NS n (%)  No.8 | p value |
| --- | --- | --- | --- |
| Male | 4 (80) | 4 (50) | 0.56 |
| Age | 58.6± 11 | 60.8±9.8 | 0.2 |
| sO2 | 98± 1.4 | 71± 10.8 | **0.04** |
| Diabetes | 1 (20) | 5 (62.5) | 0.26 |
| Cardiovascular diseases | 0 | 3 (37.5) | 0.23 |
| Cancer | 0 | 0 | NA |
| Asthma | 1 (20) | 1 (12.5) | 1 |
| Insulin | 1 (20) | 0 | 0.3 |
| Anticoagulant | 1 (20) | 3 (37.5) | 1 |
| Steroids | 1 (20) | 5 (62.5) | 0.26 |
| Hydroxychloroquine | 0 | 0 | - |
| IL6 inhibitors | 1 (20) | 1 (12.5) | 1 |
| Remdesivir | 0 | 4 (50) | 0.1 |
| Ivermectin | 0 | 1 (12.5) | 1 |
| Carbapenem antibiotics | 2 (40) | 5 (62.5) | 0.5 |
| Fluoroquinolone | 0 | 2 (25) | 0.4 |
| Oxazolidinone antibiotic | 0 | 0 | - |

Most variables showed no statistically significant differences between ICU survivors and non-survivors. The only significant finding was a lower mean oxygen saturation (sO₂) among non-survivors (71 ± 10.8) compared to survivors (98 ± 1.4) (*p* = 0.04). Other parameters, including age, sex, comorbidities, and treatment regimens, were comparable between the two groups (*p* > 0.05).

Fluorescent Imaging Mitosox

**Table 25. Patients’ Laboratory Results.**

|  | ICU-S n (%)  No. 5 | ICU-NS n (%)  No.8 | P-value |
| --- | --- | --- | --- |
| WBCs (×10^3^ /ml) | 9.5± 3.2 | 14.3± 5.2 | 0.2 |
| Platelets (10^6^ /ml) | 240 (160-321) | 160 (80-160) | 0.4 |
| Lymphocytes | - | - | - |
| Monocytes | - | - | - |
| INR | 1± 0.01 | 1.2± 0.2 | 0.16 |
| CRP (mg/L) | 104.8± 54.6 | 119.3± 89 | 0.5 |
| D**-**Dimer (mg/ml) | 1.8± 1.7 | 4± 3.5 | 0.2 |
| IL-6 (pg/ml) | - | - | - |
| Ferritin | 221 (218-439) | 1185 (139- 2000) | 0.18 |
| Albumin | 2.9± 0.9 | 2.2± 0.4 | 0.6 |
| Hemoglobin (g/dl) | 11.7± 2 | 11.5± 2.7 | 0.8 |
| ALT (U/L) | - | - | - |
| AST (U/L) | - | - | **-** |
| Creatinine (mg/dl) | 1.6 (1-1.19) | 1.5 (1-5.6) | 0.8 |

No statistically significant differences were found between ICU survivors and non-survivors in any of the assessed parameters (*p* > 0.05). However, ferritin levels were notably higher in non-survivors compared to survivors (1185 vs. 221 ng/ml), suggesting a trend toward significance (*p* = 0.18). Other laboratory markers, including WBCs, CRP, D-dimer, albumin, and creatinine, were comparable between the two groups.

**Supplementary Table 26. Controls Demographics**

| Controls | Age | Gender |
| --- | --- | --- |
| C1 | 25 | F |
| C2 | 24 | M |
| C3 | 27 | F |
| C4 | 32 | F |
| C5 | 28 | M |
| C6 | 28 | M |
| C7 | 55 | M |
| C8 | 26 | F |
| C10 | 55 | M |
| C11 | 32 | M |
| C12 | 36 | M |
| C13 | 36 | M |
| C14 | 55 | M |
| C15 | 40 | M |
| C16 | 40 | M |
| C17 | 35 | F |
| C18 | 31 | M |
| C19 | 36 | M |
| C20 | 32 | M |
| C21 | 45 | M |
| C22 | 28 | F |
| C23 | 32 | M |
| C25 | 25 | F |
| C26 | 27 | F |
| C27 | 28 | F |
| C29 | 41 | F |
| C30 | 19 | M |
| C31 | 35 | M |
| C32 | 28 | M |

**Supplementary Table 27: Sample size per technique**

| **Technique / analysis** | **Location** | **Controls** | **ICU-S** | **ICU-NS** |
| --- | --- | --- | --- | --- |
| Clinical cohort (overall ICU patients) | Methods / cohort | — | 36 | 58 |
| Flow cytometry (whole blood) | Neutrophil proportion (Fig 1) | 27 | 32 | 48 |
| Flow cytometry (whole blood) | Neutrophil maturity CD16hi/CD16lo (Fig 1) | 10 | 16 | 15 |
| Flow cytometry | Annexin V Pacific Blue (Fig 1) | 9 | 5 | 18 |
| Flow cytometry | Annexin V FITC (Fig 1) | 13 | 8 | 7 |
| Fluorescence imaging (isolated neutrophils) | Annexin V intensity (Fig 1) | 7 | 6 | 11 |
| miRNA sequencing (purified neutrophils) | miRNA-seq (Fig 2) | — | 2 | 4 |
| Immunofluorescence imaging (isolated neutrophils) | Cleaved caspase-3 (Fig 2) | 5 | 2 | 5 |
| Flow cytometry (whole blood) | Cytosolic Ca²⁺ Fluo-4 MFI (Fig 3) | 23 | 16 | 28 |
| Fluorescence imaging (isolated neutrophils) | Fluo-4 intensity (Fig 3) | 8 | 5 | 9 |
| Immunofluorescence imaging (isolated neutrophils) | MICU1 (Fig 3) | 5 | 2 | 7 |
| Immunofluorescence imaging (isolated neutrophils) | Cyclophilin D (Fig 3) | 6 | 2 | 7 |
| Flow cytometry (whole blood) | ΔΨm TMRM MFI (Fig 4) | 23 | 16 | 28 |
| Flow cytometry (whole blood) | TMRM-high % (Fig 4) | 22 | 16 | 28 |
| Flow cytometry (whole blood) | TMRM-low % (Fig 4) | 23 | 16 | 28 |
| Seahorse XF (isolated neutrophils) | Maximal respiration (Fig 4) | 7 | 15 | 16 |
| High-resolution respirometry (isolated neutrophils) | Basal OCR (Fig 4) | 15 | 10 | 28 |
| High-resolution respirometry (isolated neutrophils) | Complex II OCR (Fig 4) | 14 | 10 | 28 |
| High-resolution respirometry (isolated neutrophils) | Complex I OCR (Fig 4) | 14 | 9 | 28 |
| TEM | Mitochondria per neutrophil (Fig 5) | 4 | 5 | 4 |
| Fluorescence imaging (isolated neutrophils) | MitoTracker (Fig 5) | 3 | 5 | 10 |
| Fluorescence imaging (isolated neutrophils) | MitoSOX (Fig 5) | 12 | 5 | 9 |
